# Transcriptional corepressors in maize maintain meristem development

**DOI:** 10.1093/plphys/kiae476

**Published:** 2024-09-10

**Authors:** Jason Gregory, Xue Liu, Zongliang Chen, Cecilia Gallardo, Jason Punskovsky, Gabriel Koslow, Mary Galli, Andrea Gallavotti

**Affiliations:** Waksman Institute of Microbiology, Rutgers University, Piscataway, NJ 08854-8020, USA; Department of Plant Biology, Rutgers University, New Brunswick, NJ 08901, USA; Waksman Institute of Microbiology, Rutgers University, Piscataway, NJ 08854-8020, USA; Waksman Institute of Microbiology, Rutgers University, Piscataway, NJ 08854-8020, USA; Waksman Institute of Microbiology, Rutgers University, Piscataway, NJ 08854-8020, USA; Waksman Institute of Microbiology, Rutgers University, Piscataway, NJ 08854-8020, USA; Waksman Institute of Microbiology, Rutgers University, Piscataway, NJ 08854-8020, USA; Waksman Institute of Microbiology, Rutgers University, Piscataway, NJ 08854-8020, USA; Waksman Institute of Microbiology, Rutgers University, Piscataway, NJ 08854-8020, USA; Department of Plant Biology, Rutgers University, New Brunswick, NJ 08901, USA

## Abstract

The formation of the plant body proceeds in a sequential postembryonic manner through the action of meristems. Tightly coordinated meristem regulation is required for development and reproductive success, eventually determining yield in crop species. In maize (*Zea mays*), the RAMOSA1 ENHANCER LOCUS2 (REL2) family of transcriptional corepressors includes four members, REL2, RELK1 (REL2-LIKE1), RELK2, and RELK3. In a screen for *rel2* enhancers, we identified shorter double mutants with enlarged ear inflorescence meristems (IMs) carrying mutations in *RELK1*. Expression and genetic analysis indicated that *REL2* and *RELK1* cooperatively regulate ear IM development by controlling genes involved in redox balance, hormone homeostasis, and differentiation, ultimately tipping the meristem toward an environment favorable to expanded expression of the *ZmWUSCHEL1* gene, which encodes a key stem-cell promoting transcription factor. We further demonstrated that *RELK* genes have partially redundant yet diverse functions in the maintenance of various meristem types during development. By exploiting subtle increases in ear IM size in *rel2* heterozygous plants, we also showed that extra rows of kernels are formed across a diverse set of F1 hybrids. Our findings reveal that the REL2 family maintains development from embryonic initiation to reproductive growth and can potentially be harnessed for increasing seed yield in a major crop species.

## Teaser

Transcriptional corepressors that regulate hormonal and chemical cues to prevent expanded expression of a key stem cell promoting factor in maize inflorescence meristems may be used to increase seed yield.

## Introduction

The meristem is an organized structure containing a pool of stem cells, which is the product of evolutionary innovation by plants to carefully coordinate multicellular growth during the early colonization of land (Niklas and Tiffney 2023). The formation of the shoot system by the shoot apical meristem (SAM) occurs through the repetitive production of leaf and bud primordia, resulting in a succession of phytomers. To achieve this, the meristem is organized into distinct functional zones. The central zone contains the stem cell niche; in the peripheral zone a combination of anticlinal and periclinal divisions ensures a balance of daughter cells that either remain in the niche or enter differentiation into lateral primordia; cells in the rib zone provide the initials for the differentiating ground and vascular tissues of the stem. Sandwiched between the central zone and the rib zone is the organizing center (OC), which contains a small population of cells that express the stem-cell promoting transcription factor (TF) WUSCHEL (WUS) (Mayer et al. 1998). Our understanding of the molecular mechanisms involved in meristem regulation began with the elucidation of the CLAVATA (CLV)-WUS pathway in Arabidopsis (*Arabidopsis thaliana*) (Schoof et al. 2000; Kitagawa and Jackson 2019). This negative feedback loop maintains a stable number of stem cells in the central zone ensuring a balance between stem cells and differentiating cells for proper growth. The CLV-WUS pathway involves the transcription of *WUS* in the OC from which it noncell autonomously activates the central zone specific expression of the *CLAVATA3* (*CLV3*) gene, whose product is processed into a short peptide that is perceived by LEUCINE RICH REPEAT (LRR) RECEPTOR-LIKE (LRR-RLP) and LRR-RECEPTOR-LIKE KINASE (RLK) proteins such as CLV1 in OC cells to repress *WUS* expression (Lenhard and Laux 2003) and prevent stem cell over-proliferation in the central zone of the meristem (Guo et al. 2010).

In maize (*Zea mays*), orthologous components of the CLV signaling pathway such as THICK TASSEL DWARF1 (TD1) (Bommert et al. 2005), and FASCIATED EAR2 (FEA2) (Taguchi-Shiobara et al. 2001) have been identified. The maize genome contains two co-orthologs of *WUS*, *ZmWUS1*, and *ZmWUS2* (Nardmann and Werr 2006), and recent characterization of the *Barren inflorescence3* (*Bif3*) mutant (Chen et al. 2021) demonstrated that ZmWUS1 promotes stem cell fate in the ear IM. FEA2 forms two distinct receptor complexes capable of perceiving two distinct peptides: when complexed with the G protein signaling component COMPACT PLANT2, FEA2 can perceive the CLV3 ortholog CLE7; alternatively, when complexed with the pseudokinase CORYNE (CRN), FEA2 perceive the differentiating primordia derived ligand FON2-LIKE CLE PROTEIN1 (FCP1) (Je et al. 2018). FCP1 can also repress *ZmWUS1* by the receptor FASCIATED EAR3 (FEA3), preventing its expression in the rib zone (Je et al. 2016). In a parallel signaling pathway, the bZIP TF FASCIATED EAR4 (FEA4) promotes differentiation in the peripheral zone by positively regulating genes controlling axillary meristem (AM) identity, determinacy, and auxin signaling (Pautler et al. 2015). In line with this, auxin operates antagonistically to cytokinin in the meristem, and promotes differentiation (Galli et al. 2015). As a negative regulator of inflorescence meristem (IM) development, the ratio of FEA4's less active monomeric versus active dimeric state is controlled by the redox environment. Loss of function in GLUTAREDOXINs (GRXs) result in an enrichment of oxidized, dimeric FEA4 in the meristem leading to stronger repressive activity and smaller ears (Yang et al. 2021). Similarly, in Arabidopsis the reactive oxygen species (ROS) superoxide (O_2_^−^) is enriched in the stem cells of the central zone and positively affects *WUS* expression, whereas hydrogen peroxide (H_2_O_2_) is enriched in the peripheral zone to promote differentiation. Shifting the O_2_^−^:H_2_O_2_ balance in the SAM alters *WUS* expression, ultimately increasing or decreasing SAM size (Zeng et al. 2017). Given the breadth in regulatory control of the meristem, chemical and receptor complex mediated downstream signaling may represent only a portion of the mechanisms employed by plants to ensure proper homeostasis of various meristems.

Mutants of the maize transcriptional corepressor REL2 display pleiotropic vegetative and reproductive phenotypes such as defective AM initiation and IM maintenance (Gallavotti et al. 2010; Liu et al. 2019). Transcriptional corepressors function as molecular bridges between TFs, and MEDIATOR subunits and histone modifiers to silence transcription of downstream target genes (Leydon et al. 2021). REL2 is a functional homolog of the Arabidopsis TOPLESS (TPL) protein, an essential regulator of development (Long et al. 2002; Long et al. 2006; Szemenyei et al. 2008; Smith and Long 2010). TPL is recruited by WUS in the SAM to repress cell differentiation promoting genes; this interaction is essential for WUS function and lack of it fails to rescue the premature meristem termination of *wus* mutants (Kieffer et al. 2006; Causier et al. 2012). However, loss-of-function mutations in *REL2* and its rice (*Oryza sativa*) ortholog *ASP1* cause enlarged IMs (Yoshida et al. 2012; Liu et al. 2019; Suzuki et al. 2019a), an opposite phenotype to *wus* loss-of-function Arabidopsis mutants. How transcriptional corepressors mediate meristem size regulation in monocots is therefore unknown.

## Results

### Isolation and characterization of a genetic enhancer of the *rel2* mutant

**Figure 1.**
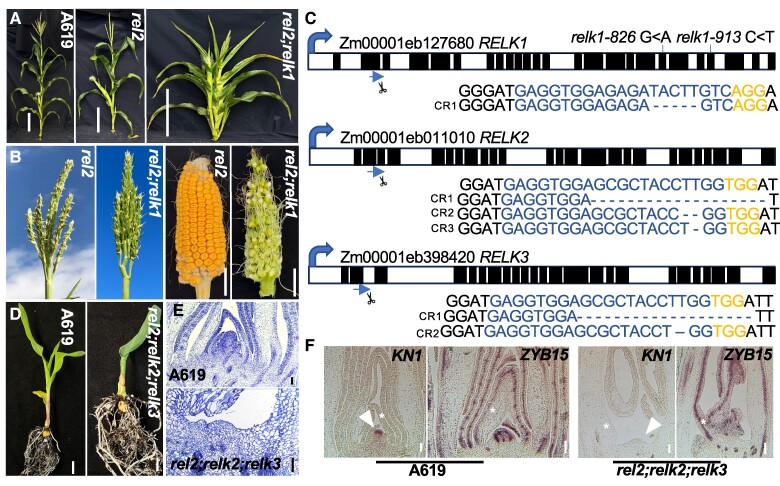
Mutants in *REL2-RELK* genes are defective in vegetative and reproductive development. **A)** Representative whole plant images of A619, *rel2*, and *rel2*;*relk1* plants. Scale bars = 30 cm. The *rel2*;*relk1* plants are smaller, with more upright tassel branches than *rel2* (B). **B)**. Ears produce few viable seeds. Scale bars = 2.5 cm. **C)** Schematic representation of the *RELK1* gene with two independent EMS alleles, *relk1-826* in exon 19 and *relk1-913* in exon 22, and one CRISPR-Cas9 generated allele using a single gRNA (blue; PAM in orange) targeted to exon 2. Alleles of *RELK2* and *RELK3* were generated using a dual targeting gRNA in exon 2. Exons in black, introns in white. **D)** By 21 days, *rel2*;*relk2*;*relk3* has only formed a single visible leaf before apical activity terminates, whereas A619 is forming new leaves. Scale bar = 1 cm. **E)** Loss of apical activity corresponds to the presence of a SAM-like remnant in seedling sections stained with toluidine-blue (scale bar = 100 μm). Arrowheads point to the SAM or SAM remnants, while asterisks point to leaf primordia. **F)** Triple mutants lack detectable *KNOTTED1* (*KN1*) (scale bar = 100 μm) but not *ZYB15* (scale bars = 200 μm) expression in RNA in situ hybridizations. Wild type sections **F)** display a normal SAM (scale bar = 100 μm), with *KN1* and *ZYB15* expression. *n* = 2–3 per genotype per experiment.

To identify genetic modifiers of the *rel2* mutant phenotype, we carried out an EMS mutagenesis screen of the *rel2-ref* allele in the permissive A619 background (Liu et al. 2019; Liu et al. 2021). We identified two M2 families (M2-01-826 and M2-01-913) that were segregating recessive mutants showing an identical phenotype, namely plants with shorter stature bearing tassels with upright tassel branches (Fig. 1A,B). We provisionally called these mutants *small upright-826* (*sup-826*) and *sup-913*. Crosses between *sup-826* and *sup-913* failed to complement the mutant phenotype in the F1 generation, indicating that the two loci carried different mutations in the same gene. We determined that the small upright phenotype was segregating with a ratio of 1:16 in F2 mapping populations (*sup-826*, 14 mutants out of 222 plants; *χ*^2^*P* = 0.95-0.99), indicating that the modifier locus was a true enhancer and did not show an obvious phenotype in a nonsensitized background. We carefully analyzed the phenotype after a few rounds of back-crosses to the original A619 background to remove unlinked EMS generated changes. Double mutant plants were characterized by a severe reduction in overall height (∼40% reduction; Supplementary Fig. S1). The reduction in stature was mainly due to a large decrease in internode length, which were approximately half the size when compared to wild type controls, as well as to a reduced number of internodes (on average 1.7 fewer internodes; Supplementary Fig. S1). Inflorescence development was also affected: double mutant tassels tended to be smaller in size (22.5 vs. 30 cm) with a similar number of branches to control plants, while ears were formed on the lowest nodes (first or second node from the brace root node; Fig. 1A; Supplementary Fig. S1). Approximately 50% of the double mutant plants did not produce an ear (12/25 plants), a phenotype reminiscent of the *rel2* phenotype in the B73 and other genetic backgrounds (Liu et al. 2019).

### The *small upright* phenotype is caused by mutations in the transcriptional corepressor RELK1

**Figure 2.**
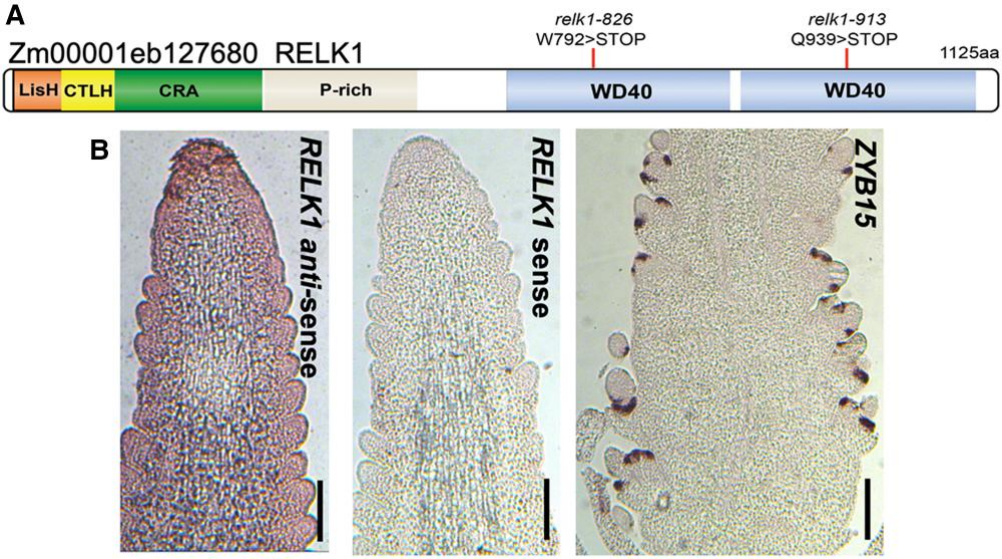
*RELK1* encodes a transcriptional co-repressor. **A)** Schematic representation of RELK1 protein domains with two independently isolated EMS alleles and corresponding mutations indicated. The *relk1-826* results in a truncation within the first WD40 repeat domain, while *relk1-913* results in a truncation within the second WD40 repeat domain. **B)** In situ hybridization in A619 wildtype ear primordia show ubiquitous expression in *RELK1* antisense but not *RELK1* sense or *ZYB15* probe treatments (*n* = 2 experimental replicates). Scale bars = 200 μm.

Double mutant *sup* plants were crossed to the inbred line B73 to create F2 mapping populations for positional cloning purposes (Gallavotti and Whipple 2015). We performed whole genome sequencing on the double mutant bulks (14 samples for *sup-826* and 18 samples for *sup-913*). In both cases, the mutant mapped on chromosome 3 and SNP analysis identified in each mutant bulk sample a base change in the coding sequence of gene model *Zm00001eb127680* (B73v5), causing premature stop codons in amino acids W792 and Q939 in *sup-826* (transition G to A) and *sup-913* (transition C to T), respectively (Fig. 1C). *Zm00001eb127680* corresponds to *RELK1*, another member of the *REL2* family of transcriptional corepressor (Liu et al. 2019). The corepressor proteins of the REL2 family are homologs of the Arabidopsis TPL/TPR family and contain conserved protein–protein interaction domains, including the N-terminal located LISH and CTLH domain and two blocks of WD40 repeats that are predicted to form a clamp-like structure (Szemenyei et al. 2008; Ke et al. 2015; Martin-Arevalillo et al. 2017). Both mutations truncated the first and second block of WD40 repeats, respectively (Fig. 2A). In Liu et al. (2019), we reported that *RELK1* was upregulated in *rel2-ref* immature tassels while the expression levels of the other remaining family members, *RELK2* and *RELK3*, remained unchanged. RELK2 and RELK3 are phylogenetically related to AtTPL and belong to the same clade (Liu et al. 2019). These results suggest that an active compensation mechanism exists specifically between *REL2* and *RELK1* and agrees with the enhancement of the *rel2* phenotype in *sup* mutants. We confirmed by RT-qPCR that the increased levels of expression of *RELK1* were also observed in young vegetative tissue of *rel2-ref* mutants (Supplementary Fig. S2). From here thereafter, we will refer to *sup* mutants as *rel2;relk1* double mutants.

The *RELK1* gene is expressed in every maize tissue (Liu et al. 2019) and we verified its expression pattern by in situ hybridizations in immature ears (Fig. 2B). A broad expression pattern of *RELK1* appeared in every cell similar to what was originally reported for *REL2* (Gallavotti et al. 2010).

### Triple *rel2;relk2;relk3* mutants fail to maintain vegetative development

**Figure 3.**
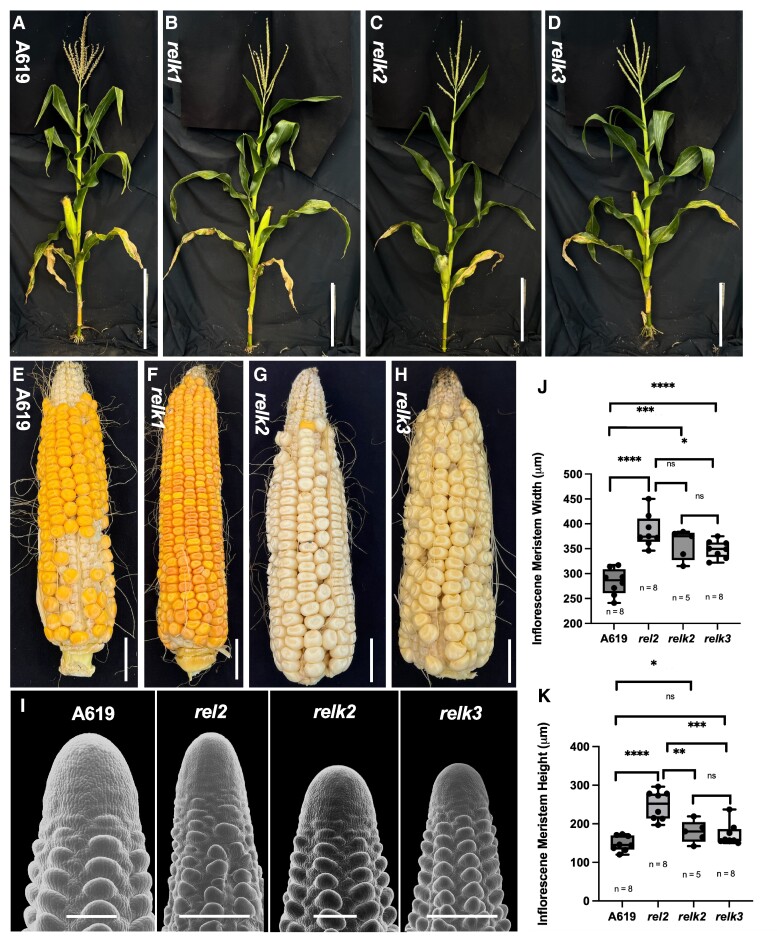
The *relk* mutants are phenotypically normal. Relative to A619 **A)**, mature field grown plants of **B)***relk1*, **C)***relk2*, and **D)***relk3* are phenotypically WT. Scale bar = 30 cm. **E–H)** Mature ears of *relk1*, *relk2*, and *relk3* are similar in size and seed yield to wild type. Scale bar = 2.5 cm. **I–K)** Similar to *rel2*, *relk2*, and *relk3* have a significant increase in IM width without the concomitant increase in IM height. In **I**) SEM images were digitally extracted for comparison, Scale bar = 100 μm. Quantification by two-tailed Student's *t*-test. Box plot center line corresponds to median; box limits, upper and lower quantiles; whiskers, maximum and minimum values. ns = nonsignificant (*P* > 0.05), * = *P* ≤ 0.05, ** = *P* ≤ 0.01, ***P ≤ 0.001, ****P ≤ 0.0001.

**Figure 4.**
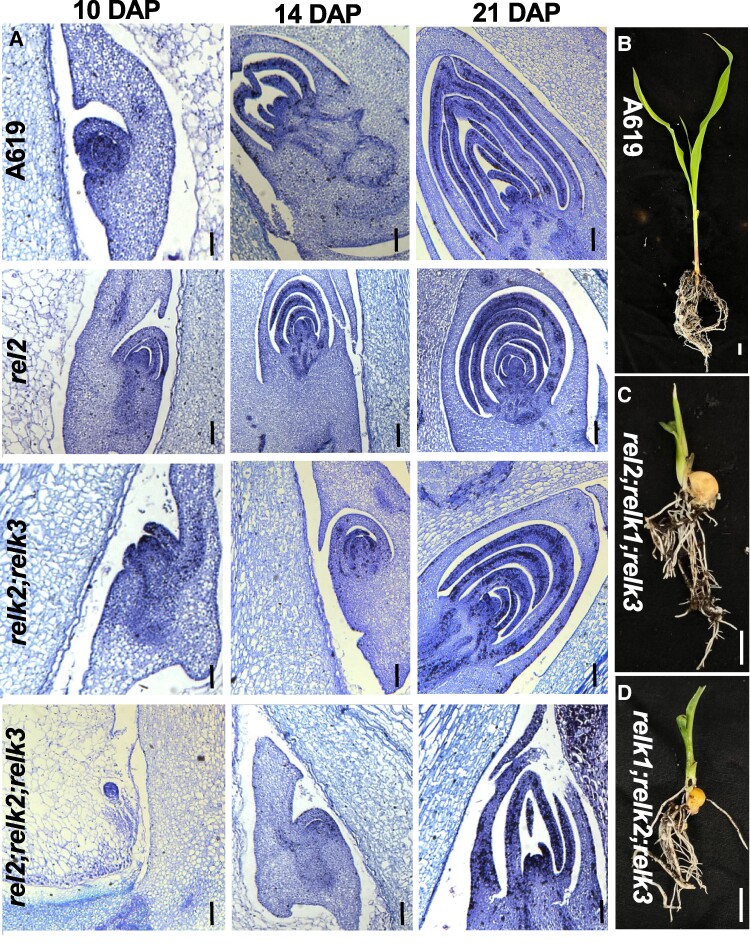
Higher order *rel2-relk* mutants show defects in SAM maintenance during embryogenesis. **A)** Defects in SAM formation begin as early as 10 DAP in *rel2*;*relk2*;*relk3* with defects in maintenance extending 14 DAP and 21 DAP. While SAM maintenance defects were detected in *relk2*;*relk3* 10 DAP, double mutants were able to recover SAM activity. By 10 DAP, *rel2* formed a SAM and two leaf primordia similar to A619 and carried out normal embryo development 14 DAP and 21 DAP analogous to A619. *n* = 2–3 per genotype, except 21 DAP *rel2*;*relk2*;*relk3 n* = 1. Scale bars, 100 μm. **B–D)** To explore whether loss of apical activity was unique to *rel2*;*relk2*;*relk3*, **C)***rel2*;*relk1*;*relk3*, and **D)***relk1*;*relk2*;*relk3* triple mutants were developed. Whereas by 20–25 days, **B)** A619 has formed a series of leaves, apical activity in the triple mutants terminates after the formation of two leaves similar to *rel2*;*relk2*;*relk3*. Scale bar = 1 cm.

As mentioned above, *RELK2* and *RELK3* are two paralogous gene that belong to the TPL clade (Liu et al. 2019). To investigate their function, we used CRISPR-Cas9 to induce mutations in both genes, which share 94% sequence identity, with one gRNA targeting their second exon. We functionally characterized a combination containing a 16 bp deletion in both genes (Fig. 1C). Double *relk2;relk3* mutant, as well as single *relk1*, *relk2*, and *relk3* mutants did not show any major developmental defects (Fig. 3 and Supplementary Fig. S3). However, when crossed to either the *rel2-203* or *rel2-ref* allele triple mutant plants were severely impaired in shoot development (Fig. 1D; Supplementary Fig. S3). In the majority of progenies of +/*rel2;relk2;relk3* self-pollinated plants, we observed a skewed 1:1 segregation for *relk2;relk3* and *+/rel2;relk2;relk3* indicating that most triple mutant gametes did not transmit regularly through generations. Occasionally, some *rel2;relk2;relk3* plants developed but the majority of plants failed to develop seedling leaves or when formed appeared as fused and tubular structures (Fig. 1D). Only a few rare plants made it to the reproductive phase, but those plants carried tassels with no flowers and no ears were formed (Supplementary Fig. S3). Crosses of additional alleles of *relk2* and *relk3* with *rel2-203* or *rel2-ref* recapitulated these phenotypes (Supplementary Fig. S3) that are reminiscent of the original temperature-sensitive *tpl1-1* allele of Arabidopsis (Long et al. 2006). Indeed, when sectioned, these plants show a lack of formation and maintenance of the SAM. Relative to the A619 SAM, the triple mutant SAM appears to be missing (Fig. 1E). Correspondingly, we verified loss of meristem identity by in situ hybridizations with the meristem marker *KNOTTED1* (*KN1*). Whereas, *KN1* expression was detected in the A619 SAM, this expression was absent in the triple mutant SAM remnants (Fig. 1F). In both genotypes, we detected strong expression of the control marker *ZYB15* (Fig. 1F) suggesting lack of *KN1* expression in triple mutants was not due to technical issues. We also sectioned developing embryos at three developmental time points after pollination (days after pollination, DAP) (Fig. 4). By 10 DAP, wild-type and *rel2* embryos had formed the SAM and several leaf primordia while in *relk2;relk3* the SAM appeared still exposed (Fig. 4); however, in *rel2;relk2;relk3* no SAM formation occurred at this stage, and *rel2;relk2;relk3* mutants were still at the proembryo globular stage. By 14 DAP (Fig. 4) *rel2;relk2;relk3* embryos were able to form a SAM-like structure, but at 21 DAP *rel2;relk2;relk3* embryos had formed fewer leaf primordia compared to the other samples, and the SAM appeared flattened suggesting that halfway through embryo development the SAM was not maintained (Fig. 4); *relk2;relk3* was indistinguishable relative to wild-type embryos by 21 DAP (Fig. 4), when no visible differences were observed. These zygotic developmental defects, as well as potential gametophytic effects may explain the skewed 1:1 segregation for +/+;*relk2;relk3* and *+/rel2;relk2;relk3* in the majority of progenies of +/*rel2;relk2;relk3* self-pollinated plants (Supplementary Fig. S3). In order to understand whether this phenotype was due to functional diversification or a higher order effect we generated *rel2;relk1;relk3* and *relk1;relk2;relk3* mutants, which phenocopied the *rel2;relk2;relk3* termination phenotype (Fig. 4). Overall, these results indicate that *REL2*, *RELK1*, *RELK2*, and *RELK3* function redundantly during embryogenesis and during shoot development postgermination.

To investigate possible functional diversification and redundancy among *RELK* genes, we also generated *rel2;relk2* and *rel2;relk3* double mutants. Interestingly, unlike *rel2;relk1* mutants, *rel2;relk2* and *rel2;relk3* plants reached a height similar to wild type plants. A few differences in average internode length and internode numbers relative to *rel2* were observed: for example, we found a statistically significant decrease in average internode length of *rel2;relk3* relative to A619, but this was compensated by the formation of an additional one or two internodes in the double mutant (Supplementary Fig. S4). The *rel2;relk2* mutants produced fewer tassel branches relative to *rel2;relk3*; however, there were overall nonsignificant differences when compared to A619 and *rel2* (Supplementary Fig. S4). While all three double mutants had similar upright tassel branches (Supplementary Fig. S4), we observed differences in ear development such that 100% of *rel2;relk3* mutants failed to produce ears, while similar to *rel2;relk1, rel2;relk2* primary ears were borne on lower internodes relative to *rel2* (Supplementary Fig. S4). These results suggest that *RELK* genes have mostly redundant yet somewhat diverse functions in maintenance of various meristem types through the plant life cycle.

### REL2 and RELK1 regulate IM size by silencing cell-proliferative signals from various pathways

**Figure 5.**
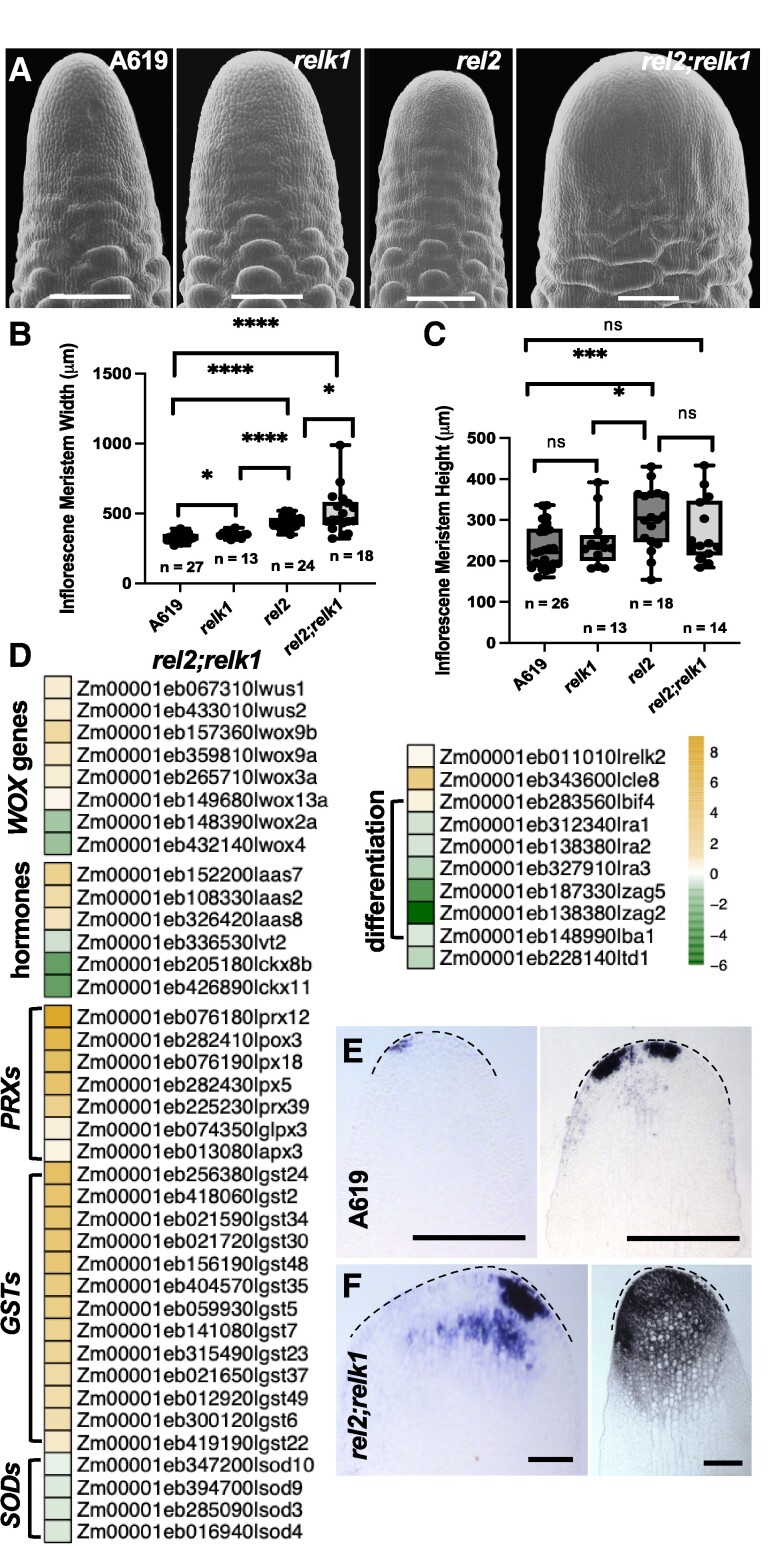
The *rel2*;*relk1* ears are fasciated. **A)** SEM images of A619, *relk1*, *rel2*, and *rel2*;*relk1* ear primordia. *rel2* ears have an increase in width **B)** leading to a cylindrically shaped IM, which becomes enhanced into a tongue shape in *rel2*;*relk1.* In A) SEM images were digitally extracted for comparison. **C)***rel2* and *rel2*;*relk1* mutants do not affect IM height. **D)** Heatmap of *rel2;relk1* DEGs in RNA-seq data (scale = LogFC) revealed misregulation of peroxidases (*PRXs*) and superoxide dismutases (*SODs*), which suggested an IM environment locally high in O_2_^−^, which was confirmed by stronger NBT blue staining of **F)***rel2*;*relk1* relative to **E)** A619 ear primordia. Black dotted lines were over imposed to highlight the IM contour. *n* = 5 per genotype. Quantification by two-tailed Student's *t*-test. Box plot center line corresponds to median; box limits, upper and lower quantiles; whiskers, maximum and minimum values; ns = nonsignificant (*P* > 0.05), * = *P* ≤ 0.05, ** = *P* ≤ 0.01, ***P ≤ 0.001, ****P ≤ 0.0001. Scale bars = 200 mm.

The most striking phenotype observed in *rel2;relk1* double mutants was a strong enhancement of the size of ear IMs (fasciation; Fig. 5A), even though ears failed to develop fully and often did not produce many seeds after fertilization (Fig. 1B). We quantified the size of the IMs and noticed that they tended to be ∼20% larger than wild type (Fig. 5B). This enhancement of ear IM size was similarly observed in *rel2;relk2* ears, which were ∼35% larger than wild-type and also produced small mature ears with few viable seeds (Supplementary Fig. S4). Interestingly, expression analysis of immature *rel2;relk2* ear primordia revealed that *RELK1* was strongly upregulated while *RELK3* was severely downregulated, suggesting that *RELK1* is not only unable to buffer against loss of *RELK2* function but may repress *RELK3* (Supplementary Fig. S5). Overall, *RELK1* appears as the compensatory gene within the *REL2* family as its expression was upregulated in vegetative tissue of *rel2*, *relk2*, and *relk3* single mutants (Supplementary Fig. S2).

To understand whether *REL2* and *RELK1* affect meristem size by influencing the core CLV-WUS pathway, we collected the first 1 mm tip of immature ears in three different genotypes, A619, *rel2-ref/rel2-ref*, and *rel2-ref/rel2-ref;relk1-826/relk1-826* mutants and profiled them by RNA-seq. Differential gene expression analysis was performed, and in *rel2* tips 1,755 genes were upregulated and 1,265 genes were downregulated while in *rel2;relk1* tips 2,585 genes were upregulated and 2,130 genes were downregulated. Between these, 889 genes were similarly upregulated and 726 genes downregulated in both mutant backgrounds (Supplementary Fig. S6). Within these common genes, there was a significant misregulation of several TF families: SBP, ARF (exclusive upregulated), EREB, DOF, WOX, AUX/IAA (predominantly upregulated), WRKY, HB, bZIP, BHLH, and MADS (equal upregulated and downregulated genes) (Supplementary Data Set 1). Interestingly, in line with genetic enhancement of IM fasciation, we found an increase in the number of genes per transcriptional factor family which were misregulated. For example, whereas only *WOX9b* was upregulated in *rel2* tips, 5 additional *WOX* genes were misregulated in *rel2 relk1* tips (Fig. 5D; Supplementary Fig. S6). Similarly, we found several upregulated genes encoding ROS scavenging enzymes including 5 and 7 PEROXIDASES (PRXs), and 7 and 13 GLUTATHIONE TRANSFERASES (GSTs), while 1 and 4 SUPEROXIDE DISMUTASES (SODs) were downregulated in our *rel2* and *rel2;relk1* data, respectively (Fig. 5D; Supplementary Fig. S6). In addition, significant changes in expression were detected in *CYTOKININ OXIDASE* (*CKX*), *AUXIN AMIDO SYNTHETASE* (*AAS*), and the *TRYPTOPHAN AMINOTRANSFERASE VT2* genes (Fig. 5D) (Phillips et al. 2011), which are involved in hormone degradation, conjugative inactivation, and synthesis, respectively. In line with this, GO annotation analysis performed on the *rel2;relk1* DEGs showed that these genes were highly enriched for biological processes related to response to chemical stimulus (GO:0044238, *P* = 2.7e-14), response to stress (GO:0006950, *P* = 7.2e-7), response to hormone stimulus (GO:009725, *P* = 0.00067), hormone-mediated signaling pathway (GO:0009755, *P* = 0.008), purine nucleoside triphosphate catabolic process (GO:0009146, *P* = 0.00027), cellular amino acid and derivative metabolic process (GO:006519, *P* = 1.3e-5), and auxin mediated signaling pathway (GO:0009734, *P* = 0.00022) (Supplementary Fig. S6). Additionally, genes related to developmental process (GO:0032502, *P* = 4.1e-7) and anatomical structure development (GO:0048856, *P* = 7.1e-6) were especially enriched and corresponded to strong downregulation of differentiation and determination-related genes responsible for different AM identities such as *RAMOSA1* (*RA1*), *RA2*, *RA3, ZAG5, ZAG2*, and *BARREN STALK1* (Mena et al. 1995; Gallavotti et al. 2004; Vollbrecht et al. 2005; Bortiri et al. 2006; Satoh-Nagasawa et al. 2006) (Fig. 5D). In order to confirm the biological relevance of misregulated ROS scavenging genes we performed NBT staining for O_2_^−^ on IM tips of A619 and *rel2;relk1* (Fig. 5E,F). We detected consistently more and darker blue staining in *rel2;relk1* tips relative to A619 corresponding to enrichment in O_2_^−^ concentration, suggesting a local increase in O_2_^−^ concentration in double mutant tips.

We also compared these results with datasets obtained in *Bif3*, a dominant mutant with enlarged ear IMs, caused by overexpression of the maize *ZmWUS1* gene (Chen et al. 2021). Only 295 differentially expressed genes (DEGs) were shared between *Bif3* and *rel2* and included genes involved in various biological processes without a discernible pattern pointing to a shared regulatory mechanism (Supplementary Data Set 2). A similar range of various processes characterized the 446 DEGs, which were shared between *Bif3* and *rel2;relk1*, and 3 genes involved in AM function (*RA2, RA3*, and *BARREN STALK1*) (Gallavotti et al. 2004; Bortiri et al. 2006; Satoh-Nagasawa et al. 2006) were downregulated in both datasets. In a comparison of *Bif3* down-*rel2* upregulated genes one notable gene was *RELK1* suggesting it is under negative regulation by *ZmWUS1.* We also compared these results with datasets obtained in *fea4,* a semi-dwarfed mutant with fasciated ears cause by mutations in a bZIP TF (Pautler et al. 2015) to see if there was overlap in DEGs between *rel2* and *fea4*. 459 DEGs were shared between *fea4* and *rel2*. In a comparison of *fea4* up*-rel2* upregulated genes, auxin related genes such as *AUX/IAAs*, and *ARFs* were enriched and this enrichment was shared in comparisons with *rel2;relk1* data (Supplementary Data Set 2). Interestingly, in a comparison of *fea4* down-*rel2* upregulated genes, a notable gene was *RELK1*, suggesting that *RELK1* is under negative regulation by *ZmWUS1* but positive regulation by *FEA4* (log2 FC −0.24).

**Figure 6.**
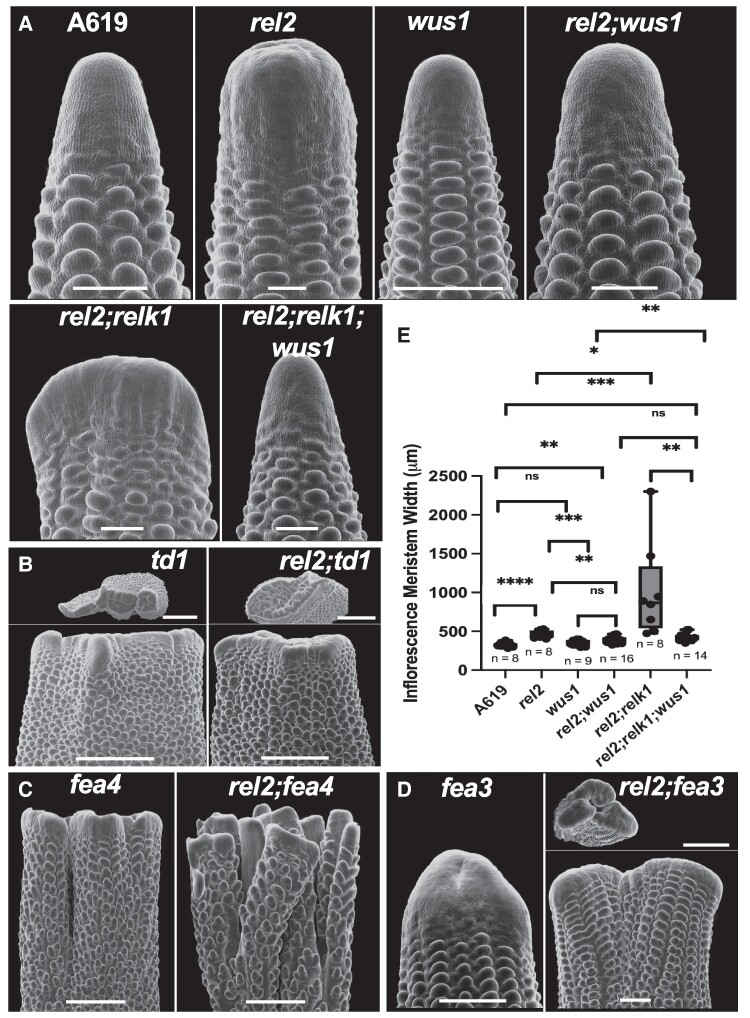
Genetic interaction with fasciated mutants. **A, E)** Introduction of a CRISPR-Cas9 generated *wus1* deletion allele into the *rel2* and *rel2*;*relk1* backgrounds rescued IM morphology and tempered but did not entirely rescue IM width relative to A619. Recovery of a conical IM in *rel2*;*relk1*;*wus1* suggests all three operate within the same pathway. **A)** Relative to *rel2*, (B) *td1* and *rel2;td1* barrel-shaped ear primordia show no discernible differences in line with an epistatic interaction. Whereas, the presence of *rel2* in the **C)***fea4* and **D)***fea3* background resulted in strong synergistic splitting of the IM relative to each single mutant. SEM images were digitally extracted for comparison. A619, *wus1* scale bar = 200 μm; *rel2*, *fea4*, *fea3*, *rel2*;*fea3*, *rel2*;*fea4*, *rel2*;*wus1* scale bar = 500 μm; *td1*, *rel2*;*td1* scale bar = 1 mm. In **B)** and **D)**, scale bars for top view = 1 mm. **E)** Quantification by two-tailed Student's *t*-test. Box plot center line corresponds to median; box limits, upper and lower quantiles; whiskers, maximum and minimum values; ns = nonsignificant (*P* > 0.05), * = *P* ≤ 0.05, ** = *P* ≤ 0.01, ***P ≤ 0.001, ****P ≤ 0.0001. SEM images, *n* = 8–16 per genotype.

To better understand the interaction of REL2/RELK corepressors with known meristem regulators, including core components of the CLV-WUS pathway, we generated *rel2;td1*, *rel2;fea4, rel2;wus1,* and *rel2;fea3* double mutants and *rel2;relk1;wus1* triple mutants. SEM analysis of immature ear primordia revealed synergistic effects between *rel2*, and *fea3* and *fea4* mutants (Fig. 6A–D). In the case of *rel2;fea3* double mutants, enlargement of the IM resulted in IM splitting, which was not detected in *fea3* single mutants (Fig. 6D) and resulted in different mature ear morphologies (Supplementary Fig. S7). In *fea4* mutants, the IM normally splits (Fig. 6C), whereas in *rel2;fea4* we observed an enhancement in the degree of splitting. Whereas, *fea4* mutants can form a mature ear with abundant seeds, *rel2;fea4* ears were smaller and failed to produce seeds (Supplementary Fig. S7). Our analysis of *rel2;td1* developing primordia and mature ears found no discernible differences between *td1* and *rel2;td1* (Fig. 6B; Supplementary Fig. S7). Occasionally, the cleft seen in the top view SEM of the ear primordia appeared wider in *rel2;td1* relative to *td1* however mature ears lack any clear differences (Supplementary Fig. S7). Overall, these data suggest an epistatic relationship between *TD1* and *REL2*, which may be in part explained by the known direct physical interaction of TPL corepressors with AtWUS. We confirmed that ZmWUS1 can physically interact with REL2 and RELK1 in Y2H assays (Supplementary Fig. S7), and may be therefore responsible for mediating ZmWUS1's repressive activity, similar to AtTPL (Causier et al. 2012). Therefore, taking advantage of previously generated CRISPR-Cas9 alleles of *ZmWUS1* (Chen et al. 2021), we also obtained *rel2;wus1* and *rel2;relk1;wus1* mutants. We found nonsignificant differences in IM size between A619 and *wus1* single mutants bearing an 8 bp deletion near the end of the DNA binding domain. In *rel2;wus1* and *rel2;relk1;wus1*, we observed a significant rescue of fasciation (Fig. 6A,E) in which the morphology of the meristem returned to a normal conical shape, but width was still significantly increased relative to A619. Take together with the upregulation of *ZmWUS1* (Fig. 5C), our data suggests that *rel2;relk1* mutants promote a molecular environment, which tips the meristem toward fasciation by expanding *ZmWUS1* expression. The absence of a complete rescue by the loss of function mutation in *ZmWUS1* indicates that other stem-cell promoting factors contribute to the fasciated phenotype, in accordance with the differential expression analysis.

### Mutations in *REL2* increase seed yield

**Figure 7.**
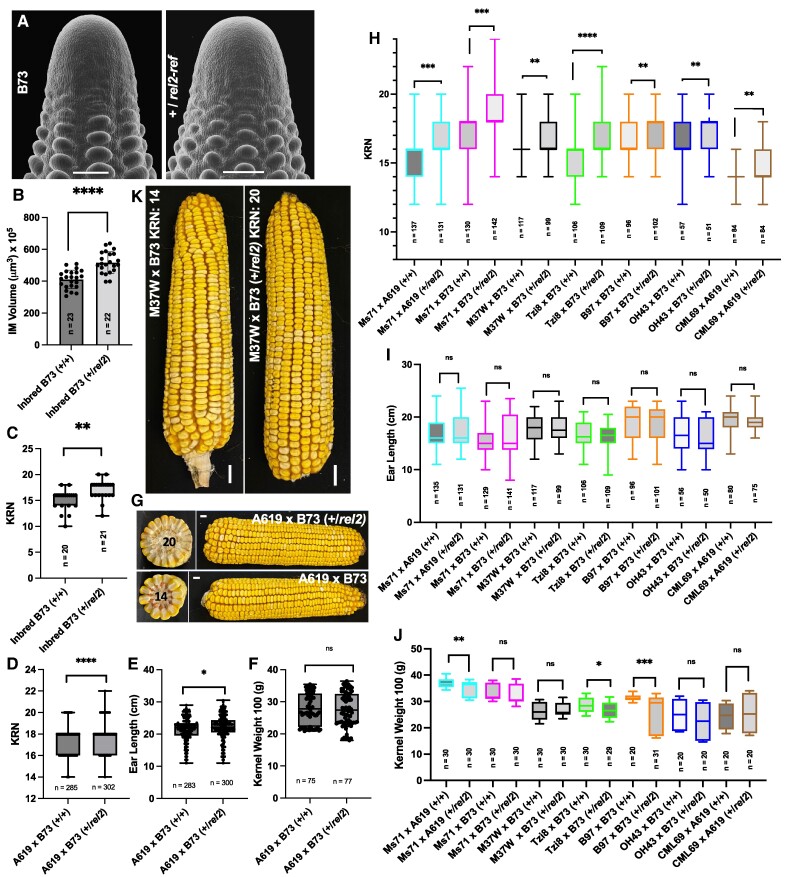
The *rel2-ref* allele increases KRN. **A)** SEM images of B73 wild-type and +/*rel2* ear primordia. Scale bars = 200 μm. SEM images were digitally extracted for comparison. The presence of a single mutant allele results in a significant increase in IM volume **B)**. Concomitantly, heterozygous mutant inbred lines **C)** and **D–G)** A619 x B73 F1's display an increase in KRN relative to wild type controls without affecting kernel weight; only a subtle increase in length was observed in +/*rel2* hybrids. **H–J)** 22 additional F1 hybrid combinations were tested for increased KRN. Of these, H) 7/22 displayed increased KRN in two to three environmental replicates without significant alterations in ear length I), though pleiotropic effects on kernel weight J) were observed. **K)** Representative image of M37W x B73 (+/+) and M37W x B73 (+/*rel2*) ears showing an increase KRN without morphological deformity of the ear. Scale bars = 1 cm. Quantification by two-tailed Student's *t*-test. Box plot center line corresponds to median; box limits, upper and lower quantiles; whiskers, maximum and minimum values; ns = nonsignificant (*P* > 0.05), * = *P* ≤ 0.05, ** = *P* ≤ 0.01, ***P ≤ 0.001, ****P ≤ 0.0001.

We previously reported that single *rel2* mutants in the permissive A619 background produced subtle increases in ear IM size and subsequently mature ears with an increase in rows of kernels, a yield trait called kernel row number (KRN) (Liu et al. 2019). In B73 however, *rel2-ref* homozygous mutants failed to develop ears (Liu et al. 2019). We subsequently noticed a similar subtle increase in IM size in *rel2-ref* heterozygous plants in the B73 inbred background (Fig. 7A,B) that produced ears with a tendency to form two extra rows of kernels (Fig. 7C). This heterozygous effect on ear size prompted us to test it in F1 hybrids, which are commonly used for seed production. We first tested the *rel2* heterozygous effect in F1 hybrids obtained by crossing A619 (+/+) and B73 (*rel2-ref/rel2-ref*). Results from three independent fields indicated that +/*rel2* F1 hybrids produced two extra rows of kernels on average (*P* < 0.0001) when compared to A619 (+/+)×B73 (+/+) hybrids (Fig. 7D,G). The size of the ears and kernel weight were not significantly affected by the *rel2* mutation, apart for a subtle increase in length (Fig. 7E,F; Supplementary Data Set 3). To thoroughly evaluate the yield performance of these and other hybrid combinations, including of elite inbred lines, we also generated 36 additional F1 hybrids, using as parents mostly a subset of the NAM founder lines, which capture different maize groups and a majority of variability existing in maize germplasm, as well as other available inbreds (Supplementary Data Set 3) (Yu et al. 2008). We carried out limited field trials in at least two independent fields, either in different years or in different locations (NJ and HI) for additional 22 of the 36 F1 hybrids. Overall, 40% of the tested hybrids with replicates produced significant increases in KRN (Fig. 7H; Supplementary Fig. S8); however, only in four additional combinations (OH43xB73, CML69xA619, M37WxB73, and Ms71xB73) were these increases observed without adversely affecting ear size (Fig. 7I) or kernel weight (Fig. 7J). Analysis of variance (ANOVA) indicated that the environment had a significant effect on the three measured traits (Supplementary Datasets S3 and S4), with a couple of exceptions (M37WxB73 and CML69xA619) showing a consistent increase in KRN. We did not observe any specific trend in hybrid combinations of specific inbred groups as defined in Flint-Garcia et al. (2005). Overall, these data suggest that heterozygosity at the *rel2* locus can potentially be used to significantly increase maize seed yield in a subset of hybrid combinations.

## Discussion

Transcriptional corepressors are master regulators of development in eukaryotes (Long et al. 2006; Payankaulam et al. 2010; Turki-Judeh and Courey 2012; Bailey et al. 2022). Previous studies from Arabidopsis, rice and maize have highlighted their importance within the Angiosperms in regulating developmental programs and immune responses (Long et al. 2006; Gallavotti et al. 2010; Pauwels et al. 2010; Zhu et al. 2010; Yoshida et al. 2012; Walley et al. 2018; Liu et al. 2019; Suzuki et al. 2019a; Darino et al. 2021; Plant et al. 2021; Huang et al. 2024). In this study, we provide a comprehensive functional analysis of the *REL2* corepressor family members and their indispensable role in maize growth. Double and triple mutant analysis of family members revealed redundant and divergent roles of individual *RELK* genes. The SAM termination and early embryogenic developmental delays in *rel2;relk2;relk3* and *relk1;relk2;relk3* indicate that REL2, RELK1, RELK2, and RELK3 function redundantly during embryogenesis and shoot development postgermination, in a similar fashion to the Arabidopsis *tpl1-1* allele, a gain-of-function dominant negative mutant (Long et al. 2006). We detected a skewed 1:1 segregation for *relk2;relk3* and *+/rel2;relk2;relk3* in the majority of progenies of +/*rel2;relk2;relk3* self-pollinated plants, which may be partially explained by developmental delays during early embryogenesis, or potential gametophytic defects; however further investigation is required to parse out whether one or both causes contribute to the observed segregation. With the switch to the reproductive phase, we have shown that *REL2, RELK1,* and *RELK2* are required for maintenance of ear IM size, while *REL2* and *RELK3* function in the initiation of the axillary buds which will form ears. Taken together, our data suggests that *REL2, RELK1, RELK2,* and *RELK3* are necessary for the initial establishment and maintenance of the embryonic SAM, but *REL2* functions as the main gene, while the other members appear dispensable and can only partially compensate for its loss.

The most intriguing phenotype was the enhanced fasciation in *rel2;relk1* relative to *rel2* ears. Understanding the mechanism of fasciation has important implications for fine-tuning yield increases. Based on our analysis, *REL2* and *RELK1* act cooperatively in the IM to repress cell proliferation in the meristem thereby ensuring homeostasis and regulating size by controlling genes involved in redox balance, hormone catabolism, and differentiation. This includes the up-regulation of several AASs and a concomitant down-regulation of CKXs and VT2 (Phillips et al. 2011). This should result in an environment low in free, biologically available auxin but high in cytokinin, an essential hormone promoting stem cell proliferation. Prior work with the *barren inflorescence* mutants has demonstrated the importance of both hormones in maintaining meristems or promoting differentiation. The semidominant *Bif3* (Chen et al. 2021) mutant causes stem cell over-proliferation in the IM due to changes in cytokinin sensitivity at the *ZmWUS1* locus, while studies of *bif2* (McSteen et al. 2007) and *Bif1, Bif4* (Galli et al. 2015) have demonstrated the vital role of auxin in establishing AMs as the ear develops. From a redox standpoint, some of the most numerous and highest DEGs corresponded to *PRXs* and *GSTs* which detoxify H_2_O_2_ (Ugalde et al. 2021). Together with the concomitant downregulation of *SODs*, the *rel2;relk1* ear IM should be scrubbed of differentiation promoting H_2_O_2_ and locally high in the stem-cell promoting O_2_^−^, a hypothesis that was supported by the NBT staining assay (Fig. 5). O_2_^−^ can be protonated to the reactive perhydroxyl radical which can initiate lipid peroxidation chains (Dat et al. 2000). GSTs function in the detoxification of H_2_O_2_ and lipid peroxides, with different enzymes bearing different affinity for one or the other (Ugalde et al. 2021). Therefore, the large number of upregulated *GSTs* may reflect not only additional means to mute differentiation but also tamper damage to cellular ultrastructure in the presence of high O_2_^−^ conditions.

Our genetic analysis between *rel2* and already characterized fasciated mutants indicates that *REL2/RELKs* mainly function in the CLV-WUS pathway, but separately from both *FEA3* and *FEA4* pathways (Fig. 6). Based on the interaction analysis of TPL and WUS in Arabidopsis, this is expected, and WUS, a bifunctional TF (Ikeda et al. 2009), is believed to work as a repressor in the OC via its physical interaction with TPL proteins and repress genes involved in differentiation (Kieffer et al. 2006; Causier et al. 2012). However, this model does not explain why loss-of-function mutations in *REL2* (and *RELK* genes) and its rice ortholog *ASP1* increase IM size, rather than decreasing it, as predicted. In rice, mutations in *TAB1*, the *WUS* ortholog, do not affect meristem size but rather the initiation of AMs (Tanaka et al. 2015; Suzuki et al. 2019a, 2019b) while in maize loss of *ZmWUS1* activity does not affect development (Chen et al. 2021), presumably due to the presence of the duplicated gene *ZmWUS2* (Nardmann and Werr 2006). However, by generating *rel2;wus1* and *rel2;relk1;wus1* mutants we showed an almost complete rescue of the fasciated ear phenotype, indicating that *ZmWUS1* overexpression observed in our RNA-seq datasets is largely responsible for the increase in meristem size. Rather than simply acting through a physical interaction with WUS proteins, our data supports a broader role for REL2-RELK in maintaining meristem homeostasis by influencing the CLV-WUS pathway, the auxin/cytokinin balance and ROS species. Overall, these factors maintain a molecular environment that tampers *ZmWUS1* expression, whose repressive activity may require REL2/RELKs.

Maize is an important direct or indirect source of food. As 30% of the world's caloric intake can be traced directly or indirectly back to maize (Shiferaw et al. 2011), even modest increases in seed yield per acre on a commercial scale can have large impacts on maintaining vital food chains. Through our hybrid analysis, we have shown the potential application of *rel2* mutants in significantly increasing maize yield. While only 20% of our F1 hybrid combinations resulted in a significant increase in KRN without detrimental pleiotropic effects, it is possible that our analysis failed to capture all potential additive variance by using only two inbred lines as sources for the *rel2-ref* allele. While these results are promising, we caution that large-scale production tests should be performed to thoroughly evaluate the yield performance of these and other hybrid combinations (Khaipho-Burch et al. 2023). Also, REL2 has been implicated in resistance to biotic stresses (Walley et al. 2018; Darino et al. 2021; Bindics et al. 2022; Huang et al. 2024); whether *rel2* heterozygous plants are more susceptible to pathogens or other stresses remains to be properly tested.

## Materials and methods

### Plant material and positional cloning of *RELK1*

Maize plants were grown in the Waksman Institute field (Piscataway, NJ, USA) or in Rutgers Hort Farm III (East Brunswick, NJ, USA) during the summer months or during the winter in the Waksman Institute greenhouse (Piscataway, NJ, USA) or in the Friendly Isle Growing Service Corporation field nursery in Hawaii (Molokai, HI, USA).

The *relk1-826* and *relk1-913* alleles were generated by EMS mutagenesis in a screen for enhancers of the *rel2-ref* mutant phenotype (Liu et al. 2021). The original mutagenesis was performed in a permissive background (A619). Both original *rel2;relk1* double mutants were then crossed to B73 to generate F2 mapping populations. A whole-genome sequencing bulked segregant analysis was subsequently performed by sequencing DNA bulk samples from pools of double mutant plants (14 *rel2;relk1-826* and 18 *rel2;relk1-913* plants). Library preparation and sequencing were performed by Macrogen/Psomagen using Illumina HiSeq X Ten and NoveSeq 6000 S4 systems, with an approximate 15 × and 50 × coverage, for *rel2;relk1-913* and *rel2;relk1-826*, respectively. Sequence analysis and SNP calling were done according to Dong et al. (2019).

To obtain CRISPR-Cas9 mutant alleles of *RELK2* and *RELK3* a dual targeting guide RNA specific for exon 2 was cloned into pBUE411 by HiFi cloning (NEB). To obtain CRISPR-Cas9 mutant alleles of *RELK1* one guide RNA specific for exon 2 was cloned into pBUE411 by HiFi cloning (NEB). The resulting construct was introduced into Hi-II immature embryos by Agrobacterium-mediated transformation. Transformation and regeneration were carried out by the Iowa State University Plant Transformation Facility. Mutant alleles were generated by crossing transgenic lines carrying CRISPR-Cas9 *RELK1;RELK2;RELK3* gRNA with A619 wild-type, *rel2-ref;relk1-826* and *rel2-203* mutant (A619) BC(2). Genotypes for single, double, and triple mutants were determined using gene-specific primers (Supplementary Table S1). For the genetic analysis, we generated double mutants by crossing *td1-mum3*, *fea4-5171, fea3-0* alleles with either *rel2-ref* (A619) or *rel2-203* (A619) (Bommert et al. 2005; Pautler et al. 2015; Je et al. 2016; Liu et al. 2019). For quantification two additional rounds of back-crosses in A619 were performed. To generate *rel2;wus1* mutants, we crossed CRISPR-Cas9 *ZmWUS1*-A specific guide RNA line (Chen et al. 2021) with *rel2-ref;relk1-826*. Quantification was carried out after three rounds of back-crosses in A619.

Field-grown *rel2-ref;relk1-826* double mutants A619 BC(6) were phenotyped for plant height (aerial root internode to tassel tip), internode number, internode length, internode at which the primary ear was formed, length of terminal internode to tassel tip, length of subtending tassel branch to tassel tip, and tassel branch number in summer 2020 and 2021 field seasons. Measurements included at least 10 samples per genotype, with significance calculated using student's two-tailed *t-*test, with a *P* value <0.05 threshold. Graphpad Prism 9 software was used to represent the data.

### Scanning electron microscopy

Freshly dissected samples of immature ears (3–5 mm) were imaged using a JMC-6000PLUS Scanning Electron Microscope within 15 min of dissection. IM width and height measurements were taken immediately after images were captured. All measurements included at least eight samples per genotype, with significance calculated using student's two-tailed *t-*test, with a *P* value <0.05 threshold. Graphpad Prism 9 software was used to represent the data. Original SEMs are presented in Supplementary Data Set 5.

### Sectioning and histology

For analysis of SAM termination, A619 wild-type and *rel2-203;relk2;relk3* seedling tissue was harvested from samples grown in the Waksman Institute greenhouse in the spring of 2021. For analysis of early embryogenesis 10 DAP, 14 DAP, 16 DAP and 21 DAP immature kernels were harvested from self-pollinations of A619 wild-type, *rel2-203, relk2;relk3,* and +/*rel2-203;relk2;relk3* ears grown in the Waksman Institute field in the summer of 2021. Immature kernels were genotyped using endosperm tissue with *rel2-203* primers (18; Supplementary Table S1). Tissues were fixed in 4% w/v PFA (paraformaldehyde; Thermo Scientific) using vacuum infiltration, then dehydrated in a graded ethanol series, treated with a graded Histoclear series (Electron Microscopy Solutions), and embedded in paraplast (Leica).

Sections (8 μm) were placed on slides (Fisherbrand), dewaxed, and stained with 0.1% w/v Toluidine Blue (in 0.6% v/v boric acid). Stained sections were rinsed with water, mounted with Permount (Electron Microscopy Solutions), and analyzed by light microscopy using a Leica DM5500B microscope equipped with a DFC450 C digital camera.

### NBT staining

Nitro blue tetrazolium staining was performed on 2–5 mm ear primordia harvested from A619 and *rel2;relk1*. Primordia were incubated in 1 mg/mL NBT solution (50 mg NBT in 50 mL 6.8 g/L KH_2_PO_4_ with 0.05% v/v Tween 20) under vacuum for 3 h and incubated overnight in the dark. Following incubation, the NBT solution was replaced with 3:1:1 ethanol:acetic acid:glycerol solution and boiled at 95°C for 15 min. Then, fixed in PFA (Thermo Scientific) dehydrated in a graded ethanol series, cleared with Histoclear (electron microscopy solutions) and embedded in Paraplast (Leica). Sections (8 μm) were placed on slides, and dewaxed. Stained sections were rinsed with water, mounted with Permount (electron microscopy solutions), and analyzed by light microscopy using a Leica DM5500B microscope equipped with a DFC450 C digital camera.

### RNA in situ hybridizations

In situ hybridization experiments were performed on 2–5 mm ear primordia and 10–21 day old seedling apexes fixed in PFA (Thermo Scientific), dehydrated in a graded ethanol series, cleared with Histoclear (Electron Microscopy Solutions) and embedded in Paraplast (Leica). Sense and antisense RNA probes for *ZmRELK1*, *ZYB15*, and *KN1* genes, using either entire/partial coding sequences or 3′-untranslated region of each gene were cloned into pGEM T-easy vectors (Promega). The plasmids were linearized by restriction endonucleases, and the sense/antisense RNA probes (with sizes ranging from 300 to 1,000 bp) were synthesized by T7 or SP6 RNA polymerase (Promega). The vectors, enzymes, and primers used for probe design are listed in Supplementary Table S1.

### RNA-seq and expression analysis

Immature ears (3–5 mm) were collected from A619 wild-type, *rel2-ref* and *rel2-ref;relk1* plants grown in winter 2021 and 2022 in the Waksman Institute greenhouse. IMs, defined as 1 mm of tissue at the tip of immature ears, were harvested per genotype per biological replicate (a total of 20–60 tips) approximately at the same time of the day (from 11 AM to 2 PM). Total RNA was isolated using Plant RNeasy Mini Kit (Qiagen). Three biological replicates per genotype were sent to Psomagen Inc. for library preparation and Illumina RNA sequencing. RNA-seq raw data were trimmed with Trimmomatic with default settings (Bolger et al. 2014). Alignment to the reference genome (B73v5 genome) was performed using HISAT 2.1.0 with default settings (Kim et al. 2015). HTSeq-count (Anders et al. 2015) was used to quantify gene expression as read counts and genes with differential expression were determined by edgeR 3.18.1 package (Chen et al. 2016). All statistical analyses of gene expression were conducted in R. Genes with a fold change *P* < 0.05 were considered as DEGs. GO Term Analysis was performed using AgriGO v2.0 GO Analysis Toolkit and Database. All RNA-seq datasets are deposited in NCBI (BioSample accession numbers SAMN40984045-SAMN40984074).

For RT-qPCR analysis, immature ears (3–5 mm) were collected from A619 wild-type, *rel2-ref, rel2;relk2,* and *rel2-ref;relk1* plants. Total RNA was extracted from three biological replicates with nine ears per replicate using Plant RNeasy Mini Kit (Qiagen). For the vegetative analysis, 1 cm of tissue (hypocotyl to leaf sheaths) were harvested from 2-week old seedlings. Total RNA was extracted from three biological replicates with three seedlings per replicated using Plant RNeasy Mini Kit (Qiagen). cDNA was obtained using the qScript cDNA Synthesis kit (Quantabio) and amplified with the Perfecta SYBR Green Fast Mix (Quantabio). RT-qPCR was performed on two biological replicates with three technical replicates using specific primer pairs for REL2 and RELK genes (Liu et al. 2019; Supplementary Table S1), and UBIQUITIN as a control. Reactions were carried out using the Illumina Eco Real-Time PCR System and quantified with the Eco Real-Time PCR System Software EcoStudy (Illumina).

### Yeast 2-hybrid

To generate the pDEST-DB-REL2, pDEST-DB-RELK1, pDEST-DB- RELK2, pDEST-DB-RELK3, and pDEST-AD-WUS1 constructs, the full-length coding sequences of REL2, RELK1, RELK2, RELK3, and WUS1 were cloned into pENTR223-Sfi and recombined into either pDEST-DB or pDEST-AD using LR clonase II (Life Technologies). pDEST-AD and pDEST-DB clones were transformed into mating compatible yeast strains Y8800 and Y8930, respectively, using the LiAc transformation method. Mating was carried out according to standard procedures. Reporter gene activation was determined by assessing growth on -Leu/-Trp/-His +1 mm 3AT media after 3 to 5 days grown at 28°C to 30°C.

### F1 hybrid analysis

The analysis of the *rel2-ref* allele was conducted with a maize panel of 23 diverse inbred lines. F1 hybrids resulting from crosses of wild-type and *rel2-ref* mutant inbred lines in the A619 and B73 backgrounds with the 22 diverse inbred lines were grown in three environments: Molokai (HI), Waksman Institute field (WIM), and Rutgers University Horticulture Farm III (HF). Crosses were performed using the *rel2-ref* mutant either as a pollen source or using its ears for A619 hybrids and as a pollen source only for B73 hybrids (*rel2-ref* is earless in B73; Liu et al. 2019). KRN, ear length, and weight of 100 kernels phenotypic data was collected from plants grown between winter 2020 and winter 2022 (raw data are available in Supplementary Data Set 3). Weight of 100 kernels was used as a proxy for seed size. For data presented in Fig. 7, statistical analysis was performed using Student's two-tailed *t-*test, with a *P*-value <0.05 threshold, using data from all replicates. Statistical analysis was also performed on these datasets using two-way ANOVA with a significance level 0.05 using data from all replicates and this analysis is presented in Supplementary Data Set 4.

Graphpad Prism 9 software was used to represent the data. Please note that in several instances the number of ears analyzed for KRN or ear length do not necessarily match; that is, this was caused by the fact that ear length could be measured but not KRN, due to poor seed set; or vice versa, KRN could be measured but not ear length because the ear broke off.

### Accession numbers

The *REL2/RELK* family genes described in this article can be found in the NCBI database under accession numbers NM_001174401.2 (*REL2*/Zm00001eb415530), MH230891 (*RELK1/*Zm00001eb127680), MH230892 (*RELK2/*Zm00001eb011010), and MH230893 (*RELK3/*Zm00001eb398420).

## Supplementary Material

kiae476_Supplementary_Data

## Supplementary data

The following materials are available in the online version of this article.

**Supplementary Figure S1.** *sup* morphology.

**Supplementary Figure S2.** Expression analysis.

**Supplementary Figure S3.** Higher order *rel2-relk* mutants.

**Supplementary Figure S4.** Functional redundancy and diversification in *rel2;relk* double mutants.

**Supplementary Figure S5.** *RELK1* but not *RELK3* buffers *rel2;relk2* ear primordia.

**Supplementary Figure S6.** Differential gene expression analysis.

**Supplementary Figure S7.** Genetic and molecular interactions.

**Supplementary Figure S8.** Additional NAM F1 Hybrids display KRN increase.

**Supplementary Table S1.** Primers used in this work.

**Supplementary Data Set 1.** RNA-seq data.

**Supplementary Data Set 2.** RNA-seq comparisons.

**Supplementary Data Set 3.** Raw data of F1 hybrid combinations.

**Supplementary Data Set 4.** ANOVA analysis.

**Supplementary Data Set 5.** Original SEMs.

## Dive Curated Terms

The following phenotypic, genotypic, and functional terms are of significance to the work described in this paper:

CLV1 Gramene: AT1G75820
CLV1 Araport: AT1G75820

SUP Gramene: AT3G23130
SUP Araport: AT3G23130

WUS Gramene: AT2G17950
WUS Araport: AT2G17950

ASP1 Gramene: AT2G30970
ASP1 Araport: AT2G30970

WUS1 Gramene: AT2G17950
WUS1 Araport: AT2G17950

NBT Gramene: Nitro blue tetrazolium
NBT Araport: Nitro blue tetrazolium

## References

[kiae476-B1] Anders S , Pyl PT, Huber W. HTSeq–a python framework to work with high-throughput sequencing data. Bioinformatics. 2015:31(2):166–169. 10.1093/bioinformatics/btu638 25260700 PMC4287950

[kiae476-B2] Bailey TB , Whitty PA, Selker EU, McKnight JN, McKnight LE. Tup1 is critical for transcriptional repression in Quiescence in S. cerevisiae. PLoS Genet. 2022:18(12):e1010559. 10.1371/journal.pgen.1010559 36542663 PMC9815585

[kiae476-B3] Bindics J , Khan M, Uhse S, Kogelmann B, Baggely L, Reumann D, Ingole KD, Stirnberg A, Rybecky A, Darino M, et al Many ways to TOPLESS—manipulation of plant auxin signalling by a cluster of fungal effectors. New Phytol. 2022:236(4):1455–1470. 10.1111/nph.18315 35944559

[kiae476-B4] Bolger AM , Lohse M, Usadel B. Trimmomatic: a flexible trimmer for illumina sequence data. Bioinformatics. 2014:30(15):2114–2120. 10.1093/bioinformatics/btu170 24695404 PMC4103590

[kiae476-B5] Bommert P , Lunde C, Nardmann J, Vollbrecht E, Running M, Jackson D, Hake S, Werr W. Thick tassel dwarf1 encodes a putative maize ortholog of the Arabidopsis CLAVATA1 leucine-rich repeat receptor-like kinase. Development. 2005:132(6):1235–1245. 10.1242/dev.01671 15716347

[kiae476-B6] Bortiri E , Chuck G, Vollbrecht E, Rocheford T, Martienssen R, Hake S. Ramosa2 encodes a LATERAL ORGAN BOUNDARY domain protein that determines the fate of stem cells in branch meristems of maize. Plant Cell. 2006:18(3):574–585. 10.1105/tpc.105.039032 16399802 PMC1383634

[kiae476-B7] Causier B , Ashworth M, Guo W, Davies B. The TOPLESS interactome: a framework for gene repression in Arabidopsis. Plant Physiol. 2012:158(1):423–438. 10.1104/pp.111.186999 22065421 PMC3252085

[kiae476-B8] Chen Y , Lun AT, Smyth GK. From reads to genes to pathways: differential expression analysis of RNA-Seq experiments using Rsubread and the edgeR quasi-likelihood pipeline. F1000Res. 2016:5:1438. 10.12688/f1000research.8987.2 27508061 PMC4934518

[kiae476-B9] Chen Z , Li W, Gaines C, Buck A, Galli M, Gallavotti A. Structural variation at the maize WUSCHEL1 locus alters stem cell organization in inflorescences. Nat Commun. 2021:12(1):2378. 10.1038/s41467-021-22699-8 33888716 PMC8062686

[kiae476-B10] Darino M , Chia KS, Marques J, Aleksza D, Soto-Jiménez LM, Saado I, Uhse S, Borg M, Betz R, Bindics J, et al Ustilago maydis effector Jsi1 interacts with Topless corepressor, hijacking plant jasmonate/ethylene signaling. New Phytol. 2021:229(6):3393–3407. 10.1111/nph.17116 33247447 PMC8126959

[kiae476-B11] Dat J , Vandenabeele S, Vranová E, Van Montagu M, Inzé D, Van Breusegem F. Dual action of the active oxygen species during plant stress responses. Cell Mol Life Sci. 2000:57(5):779–795. 10.1007/s000180050041 10892343 PMC11147059

[kiae476-B12] Dong J , Tu M, Feng Y, Zdepski A, Ge F, Kumar D, Slovin JP, Messing J. Candidate gene identification of existing or induced mutations with pipelines applicable to large genomes. Plant J. 2019:97(4):673–682. 10.1111/tpj.14153 30417446

[kiae476-B13] Flint-Garcia SA , Thuillet AC, Yu J, Pressoir G, Romero SM, Mitchell SE, Doebley J, Kresovich S, Goodman MM, Buckler ES. Maize association population: a high-resolution platform for quantitative trait locus dissection. Plant J. 2005:44(6):1054–1064. 10.1111/j.1365-313X.2005.02591.x 16359397

[kiae476-B14] Gallavotti A , Long JA, Stanfield S, Yang X, Jackson D, Vollbrecht E, Schmidt RJ. The control of axillary meristem fate in the maize ramosa pathway. Development. 2010:137(17):2849–2856. 10.1242/dev.051748 20699296 PMC2938917

[kiae476-B15] Gallavotti A , Whipple CJ. Positional cloning in maize (Zea mays subsp. mays, Poaceae). Appl Plant Sci. 2015:3(1):apps.1400092. 10.3732/apps.1400092 25606355 PMC4298233

[kiae476-B16] Gallavotti A , Zhao Q, Kyozuka J, Meeley RB, Ritter MK, Doebley JF, Pè ME, Schmidt RJ. The role of barren stalk1 in the architecture of maize. Nature. 2004:432(7017):630–635. 10.1038/nature03148 15577912

[kiae476-B17] Galli M , Liu Q, Moss BL, Malcomber S, Li W, Gaines C, Federici S, Roshkovan J, Meeley R, Nemhauser JL, et al Auxin signaling modules regulate maize inflorescence architecture. Proc Natl Acad Sci U S A. 2015:112(43):13372–13377. 10.1073/pnas.1516473112 26464512 PMC4629326

[kiae476-B18] Guo Y , Han L, Hymes M, Denver R, Clark SE. CLAVATA2 forms a distinct CLE-binding receptor complex regulating Arabidopsis stem cell specification. Plant J. 2010:63(6):889–900. 10.1111/j.1365-313X.2010.04295.x 20626648 PMC2974754

[kiae476-B19] Huang L , Ökmen B, Stolze SC, Kastl M, Khan M, Hilbig D, Nakagami H, Djamei A, Doehlemann G. The fungal pathogen Ustilago maydis targets the maize corepressor RELK2 to modulate host transcription for tumorigenesis. New Phytol. 2024:241(4):1747–1762. 10.1111/nph.19448 38037456

[kiae476-B20] Ikeda M , Mitsuda N, Ohme-Takagi M. Arabidopsis WUSCHEL is a bifunctional transcription factor that acts as a repressor in stem cell regulation and as an activator in floral patterning. Plant Cell. 2009:21(11):3493–3505. 10.1105/tpc.109.069997 19897670 PMC2798335

[kiae476-B21] Je BI , Gruel J, Lee YK, Bommert P, Arevalo ED, Eveland AL, Wu Q, Goldshmidt A, Meeley R, Bartlett M, et al Signaling from maize organ primordia via FASCIATED EAR3 regulates stem cell proliferation and yield traits. Nat Genet. 2016:48(7):785–791. 10.1038/ng.3567 27182966

[kiae476-B22] Je BI , Xu F, Wu Q, Liu L, Meeley R, Gallagher JP, Corcilius L, Payne RJ, Bartlett ME, Jackson D. The CLAVATA receptor FASCIATED EAR2 responds to distinct CLE peptides by signaling through two downstream effectors. Elife. 2018:7:e35673. 10.7554/eLife.35673 29543153 PMC5854466

[kiae476-B23] Ke J , Ma H, Gu X, Thelen A, Brunzelle JS, Li J, Xu HE, Melcher K. Structural basis for recognition of diverse transcriptional repressors by the TOPLESS family of corepressors. Sci Adv. 2015:1(6):e1500107. 10.1126/sciadv.1500107 26601214 PMC4646777

[kiae476-B24] Khaipho-Burch M , Cooper M, Crossa J, de Leon N, Holland J, Lewis R, McCouch S, Murray SC, Rabbi I, Ronald P, et al Genetic modification can improve crop yields—but stop overselling it. Nature. 2023:621(7979):470–473. 10.1038/d41586-023-02895-w 37773222 PMC11550184

[kiae476-B25] Kieffer M , Stern Y, Cook H, Clerici E, Maulbetsch C, Laux T, Davies B. Analysis of the transcription factor WUSCHEL and its functional homologue in Antirrhinum reveals a potential mechanism for their roles in meristem maintenance. Plant Cell. 2006:18(3):560–573. 10.1105/tpc.105.039107 16461579 PMC1383633

[kiae476-B26] Kim D , Langmead B, Salzberg SL. HISAT: a fast spliced aligner with low memory requirements. Nat Methods. 2015:12(4):357–360. 10.1038/nmeth.3317 25751142 PMC4655817

[kiae476-B27] Kitagawa M , Jackson D. Control of meristem size. Annu Rev Plant Biol.2019:70(1):269–291. 10.1146/annurev-arplant-042817-040549 31035828

[kiae476-B28] Lenhard M , Laux T. Stem cell homeostasis in the Arabidopsis shoot meristem is regulated by intercellular movement of CLAVATA3 and its sequestration by CLAVATA1. Development. 2003:130(14):3163–3173. 10.1242/dev.00525 12783788

[kiae476-B29] Leydon AR , Wang W, Gala HP, Gilmour S, Juarez-Solis S, Zahler ML, Zemke JE, Zheng N, Nemhauser JL. Repression by the Arabidopsis TOPLESS corepressor requires association with the core mediator complex. Elife. 2021:10:e66739. 10.7554/eLife.66739 34075876 PMC8203292

[kiae476-B30] Liu X , Bourgault R, Galli M, Strable J, Chen Z, Feng F, Dong J, Molina I, Gallavotti A. The FUSED LEAVES1-ADHERENT1 regulatory module is required for maize cuticle development and organ separation. New Phytol. 2021:229(1):388–402. 10.1111/nph.16837 32738820 PMC7754373

[kiae476-B31] Liu X , Galli M, Camehl I, Gallavotti A. RAMOSA1 ENHANCER LOCUS2-mediated transcriptional repression regulates vegetative and reproductive architecture. Plant Physiol. 2019:179(1):348–363. 10.1104/pp.18.00913 30348817 PMC6324236

[kiae476-B32] Long JA , Ohno C, Smith ZR, Meyerowitz EM. TOPLESS regulates apical embryonic fate in Arabidopsis. Science. 2006:312(5779):1520–1523. 10.1126/science.1123841 16763149

[kiae476-B33] Long JA , Woody S, Poethig S, Meyerowitz EM, Barton MK. Transformation of shoots into roots in Arabidopsis embryos mutant at the TOPLESS locus. Development. 2002:129(12):2797–2806. 10.1242/dev.129.12.2797 12050130

[kiae476-B34] Martin-Arevalillo R , Nanao MH, Larrieu A, Vinos-Poyo T, Mast D, Galvan-Ampudia C, Brunoud G, Vernoux T, Dumas R, Parcy F. Structure of the Arabidopsis TOPLESS corepressor provides insight into the evolution of transcriptional repression. Proc Natl Acad Sci U S A. 2017:114(30):8107–8112. 10.1073/pnas.1703054114 28698367 PMC5544296

[kiae476-B35] Mayer KF , Schoof H, Haecker A, Lenhard M, Jürgens G, Laux T. Role of WUSCHEL in regulating stem cell fate in the Arabidopsis shoot meristem. Cell. 1998:95(6):805–815. 10.1016/S0092-8674(00)81703-1 9865698

[kiae476-B36] McSteen P , Malcomber S, Skirpan A, Lunde C, Wu X, Kellogg E, Hake S. Barren inflorescence2 encodes a co-ortholog of the PINOID serine/threonine kinase and is required for organogenesis during inflorescence and vegetative development in maize. Plant Physiol. 2007:144(2):1000–1011. 10.1104/pp.107.098558 17449648 PMC1914211

[kiae476-B37] Mena M , Mandel MA, Lerner DR, Yanofsky MF, Schmidt RJ. A characterization of the MADS-box gene family in maize. Plant J. 1995:8(6):845–854. 10.1046/j.1365-313X.1995.8060845.x 8580958

[kiae476-B38] Nardmann J , Werr W. The shoot stem cell niche in angiosperms: expression patterns of WUS orthologues in rice and maize imply major modifications in the course of mono- and dicot evolution. Mol Biol Evol. 2006:23(12):2492–2504. 10.1093/molbev/msl125 16987950

[kiae476-B39] Niklas KJ , Tiffney BH. Viridiplantae body plans viewed through the Lens of the fossil record and molecular biology. Integr Comp Biol. 2023:63(6):1316–1330. 10.1093/icb/icac150 36316013 PMC10755189

[kiae476-B40] Pautler M , Eveland AL, LaRue T, Yang F, Weeks R, Lunde C, Je BI, Meeley R, Komatsu M, Vollbrecht E, et al FASCIATED EAR4 encodes a bZIP transcription factor that regulates shoot meristem size in maize. Plant Cell. 2015:27(1):104–120. 10.1105/tpc.114.132506 25616871 PMC4330574

[kiae476-B41] Pauwels L , Barbero GF, Geerinck J, Tilleman S, Grunewald W, Pérez AC, Chico JM, Bossche RV, Sewell J, Gil E, et al NINJA connects the co-repressor TOPLESS to jasmonate signalling. Nature. 2010:464(7289):788–791. 10.1038/nature08854 20360743 PMC2849182

[kiae476-B42] Payankaulam S , Li LM, Arnosti DN. Transcriptional repression: conserved and evolved features. Curr Biol. 2010:20(17):R764–R771. 10.1016/j.cub.2010.06.037 20833321 PMC3033598

[kiae476-B43] Phillips KA , Skirpan AL, Liu X, Christensen A, Slewinski TL, Hudson C, Barazesh S, Cohen JD, Malcomber S, McSteen P. Vanishing tassel2 encodes a grass-specific tryptophan aminotransferase required for vegetative and reproductive development in maize. Plant Cell. 2011:23(2):550–566. 10.1105/tpc.110.075267 21335375 PMC3077783

[kiae476-B44] Plant AR , Larrieu A, Causier B. Repressor for hire! the vital roles of TOPLESS-mediated transcriptional repression in plants. New Phytol. 2021:231(3):963–973. 10.1111/nph.17428 33909309

[kiae476-B45] Satoh-Nagasawa N , Nagasawa N, Malcomber S, Sakai H, Jackson D. A trehalose metabolic enzyme controls inflorescence architecture in maize. Nature. 2006:441(7090):227–230. 10.1038/nature04725 16688177

[kiae476-B46] Schoof H , Lenhard M, Haecker A, Mayer KF, Jürgens G, Laux T. The stem cell population of Arabidopsis shoot meristems in maintained by a regulatory loop between the CLAVATA and WUSCHEL genes. Cell. 2000:100(6):635–644. 10.1016/S0092-8674(00)80700-X 10761929

[kiae476-B47] Shiferaw B , Prasanna BM, Hellin J, Bänziger M. Crops that feed the world 6. Past successes and future challenges to the role played by maize in global food security. Food Secur. 2011:3(3):307–327. 10.1007/s12571-011-0140-5

[kiae476-B48] Smith ZR , Long JA. Control of Arabidopsis apical-basal embryo polarity by antagonistic transcription factors. Nature. 2010:464(7287):423–426. 10.1038/nature08843 20190735 PMC2841697

[kiae476-B49] Suzuki C , Tanaka W, Hirano HY. Transcriptional corepressor ASP1 and CLV-like signaling regulate meristem maintenance in rice. Plant Physiol. 2019a:180(3):1520–1534. 10.1104/pp.19.00432 31079034 PMC6752933

[kiae476-B50] Suzuki C , Tanaka W, Tsuji H, Hirano HY. TILLERS ABSENT1, the WUSCHEL ortholog, is not involved in stem cell maintenance in the shoot apical meristem in rice. Plant Signal Behav. 2019b:14(9):1640565. 10.1080/15592324.2019.1640565 31284830 PMC6768262

[kiae476-B51] Szemenyei H , Hannon M, Long JA. TOPLESS mediates auxin-dependent transcriptional repression during Arabidopsis embryogenesis. Science. 2008:319(5868):1384–1386. 10.1126/science.1151461 18258861

[kiae476-B52] Taguchi-Shiobara F , Yuan Z, Hake S, Jackson D. The fasciated ear2 gene encodes a leucine-rich repeat receptor-like protein that regulates shoot meristem proliferation in maize. Genes Dev. 2001:15(20):2755–2766. 10.1101/gad.208501 11641280 PMC312812

[kiae476-B53] Tanaka W , Ohmori Y, Ushijima T, Matsusaka H, Matsushita T, Kumamaru T, Kawano S, Hirano HY. Axillary meristem formation in rice requires the WUSCHEL ortholog TILLERS ABSENT1. Plant Cell. 2015:27(4):1173–1184. 10.1105/tpc.15.00074 25841039 PMC4558701

[kiae476-B54] Turki-Judeh W , Courey AJ. Groucho: a corepressor with instructive roles in development. Curr Top Dev Biol. 2012:98:65–96. 10.1016/B978-0-12-386499-4.00003-3 22305159

[kiae476-B55] Ugalde JM , Lamig L, Herrera-Vásquez A, Fuchs P, Homagk M, Kopriva S, Müller-Schüssele SJ, Holuigue L, Meyer AJ. A dual role for glutathione transferase U7 in plant growth and protection from methyl viologen-induced oxidative stress. Plant Physiol. 2021:187(4):2451–2468. 10.1093/plphys/kiab444 34599589 PMC8644736

[kiae476-B56] Vollbrecht E , Springer PS, Goh L, Buckler ESt, Martienssen R. Architecture of floral branch systems in maize and related grasses. Nature. 2005:436(7054):1119–1126. 10.1038/nature03892 16041362

[kiae476-B57] Walley JW , Shen Z, McReynolds MR, Schmelz EA, Briggs SP. Fungal-induced protein hyperacetylation in maize identified by acetylome profiling. Proc Natl Acad Sci U S A. 2018:115(1):210–215. 10.1073/pnas.1717519115 29259121 PMC5776827

[kiae476-B58] Yang RS , Xu F, Wang YM, Zhong WS, Dong L, Shi YN, Tang TJ, Sheng HJ, Jackson D, Yang F. Glutaredoxins regulate maize inflorescence meristem development via redox control of TGA transcriptional activity. Nat Plants. 2021:7(12):1589–1601. 10.1038/s41477-021-01029-2 34907313

[kiae476-B59] Yoshida A , Ohmori Y, Kitano H, Taguchi-Shiobara F, Hirano HY. Aberrant spikelet and panicle1, encoding a TOPLESS-related transcriptional co-repressor, is involved in the regulation of meristem fate in rice. Plant J. 2012:70(2):327–339. 10.1111/j.1365-313X.2011.04872.x 22136599

[kiae476-B60] Yu J , Holland JB, McMullen MD, Buckler ES. Genetic design and statistical power of nested association mapping in maize. Genetics. 2008:178(1):539–551. 10.1534/genetics.107.074245 18202393 PMC2206100

[kiae476-B61] Zeng J , Dong Z, Wu H, Tian Z, Zhao Z. Redox regulation of plant stem cell fate. EMBO J. 2017:36(19):2844–2855. 10.15252/embj.201695955 28838936 PMC5623875

[kiae476-B62] Zhu Z , Xu F, Zhang Y, Cheng YT, Wiermer M, Li X, Zhang Y. Arabidopsis resistance protein SNC1 activates immune responses through association with a transcriptional corepressor. Proc Natl Acad Sci U S A. 2010:107(31):13960–13965. 10.1073/pnas.1002828107 20647385 PMC2922275

