# Supplementary material for: Transcriptional corepressors in maize maintain meristem development
Source: Plant Physiol. 2024 Sep 10;197(1):kiae476. doi: 10.1093/plphys/kiae476 (PMC11663565; doi:10.1093/plphys/kiae476)
Supplement: kiae476_Supplementary_Data [file kiae476_supplementary_data.zip › Supplementary Figures_final.pdf]

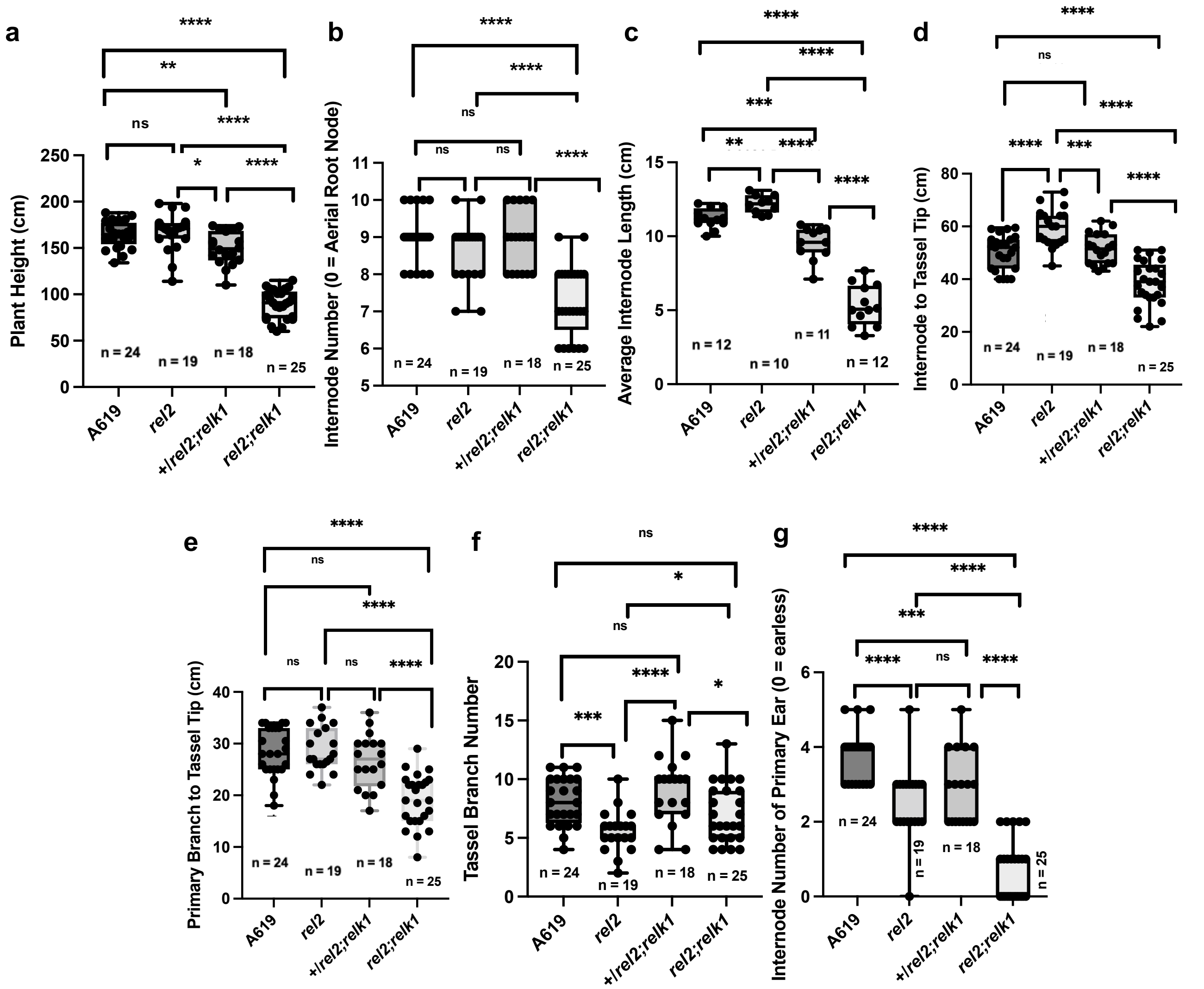

Supplemental Figure S1 *sup* morphology. *sup* morphology measurements were collected over three growing seasons on the A619 wild-type inbred line, *rel2-ref* single mutant, *+/rel2-ref;relk1* mutant, and *rel2-ref;relk1* (*sup*) double mutant**.** (**a**) Plant height. (**b**) Number of internodes, where internode 0 was defined as the node from which aerial roots were formed. (**c**) Average internode length. (**d**) Length of the tassel from terminal internode to tassel tip. (**e**) Length of the tassel first primary branch to tassel tip. (**f**) Tassel branch number. (**g**) Internode at which the primary ear developed, where 0 denotes an earless plant and internode 0 is the aerial root node. n = 10 – 25. Quantification by two-tailed Student's t-test. Box plot center line corresponds to median; box limits, upper and lower quantiles; whiskers, maximum and minimum values; ns = non-significant (p > 0.05), * = p ≤ 0.05, ** = p ≤ 0.01, *** p ≤ 0.001, **** p ≤ 0.0001.

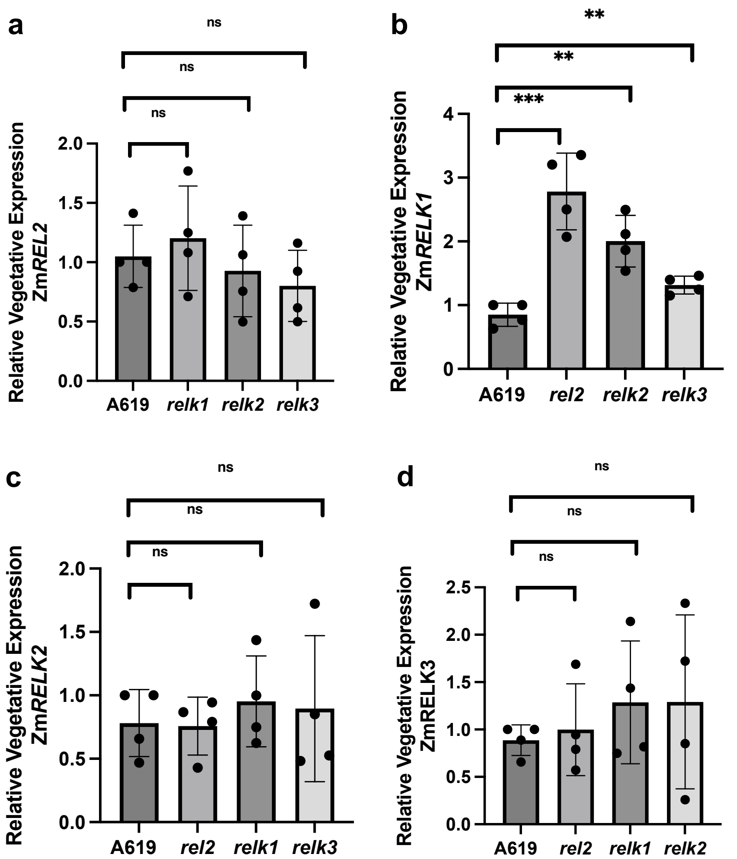

Supplemental Figure S2. Expression analysis. (**a-d**) RT-qPCR of *ZmREL2*, *ZmRELK1, ZmRELK2,* and *ZmRELK3* expression in 10-day old vegetative tissue of A619, *rel2, relk1, relk2,* and *relk3* mutants. two-tailed Student's t-test, n = 2 (pools of three seedling samples). Plot displays pooled results from two independent technical replicates. ns = non-significant (p > 0.05), * = p ≤ 0.05, ** = p ≤ 0.01, *** p ≤ 0.001, **** p ≤ 0.0001. Error bars, standard deviation.

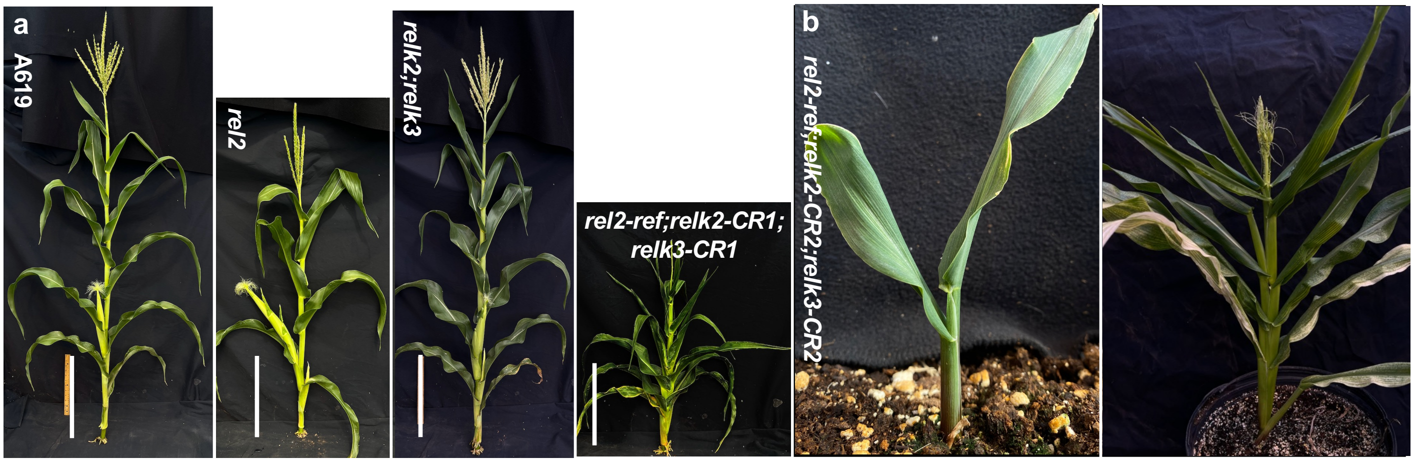

c

Year/Field/Cross	*REL2;relk2;relk3*	*+/rel2;relk2;relk3*	*rel2;relk2;relk3*
2020 WIM<br>*+/rel2-203;relk2-CR1;relk3-CR1*⊗	26/46	20/46	0 SAMT stunted seedling<br>0 SAMT plant
2021 GH<br>*+/rel2-203;relk2-CR1;relk3-CR1*⊗	13/24	10/24	1/24 SAMT stunted seedling<br>0 SAMT plant
2022 WIM<br>*+/rel2-ref;relk2-CR1;relk3-CR1*⊗	22/44	22/44	0 SAMT stunted seedling<br>0 SAMT plant
2022 WIM<br>*+/rel2-203;relk2-CR2;relk3-CR2*⊗	9/15	4/15	0 SAMT stunted seedling<br>2/15 SAMT plant
2023 GH<br>*+/rel2-ref;relk2-CR3;relk3-CR2*⊗	19/48	11/48	0/48 SAMT plant<br>18/48 non-germinated (germinated on paper)

Supplemental Figure S3. Higher order *rel2-relk* mutants. (**a**) Representative whole plant images of A619, *rel2-ref*, *relk2;relk3*, and *rel2;relk2-CR1;relk3-CR1.* A619 and *rel2-ref* whole plant images have been reused from Figure 1a. While the *rel2* single mutant displays pleiotropic vegetative and reproductive phenotypes, *relk2;relk3* is phenotypically similar to A619 wild-type. Higher order *rel2;relk2-CR1;relk3-CR1* mutant plants are shorter, and sterile. Scale bar = 30 cm. (**b**) Generation of the *rel2;relk2;relk3* triple mutant with *relk2-CR2* and *relk3-CR2* alleles phenocopies the 16bp deletion (*CR1*) allele triple mutant. (**c**) Growth of a triple mutant plant is rare, with seeds either failing to germinate or forming a stunted seedling with 1-2 leaves before apical activity terminates. Self fertilizations of +/*rel2;relk2;relk3* plants predominantly display a skewed 1(WT):1(het) segregation. GH = greenhouse growing conditions, WIM = summer field growing conditions.

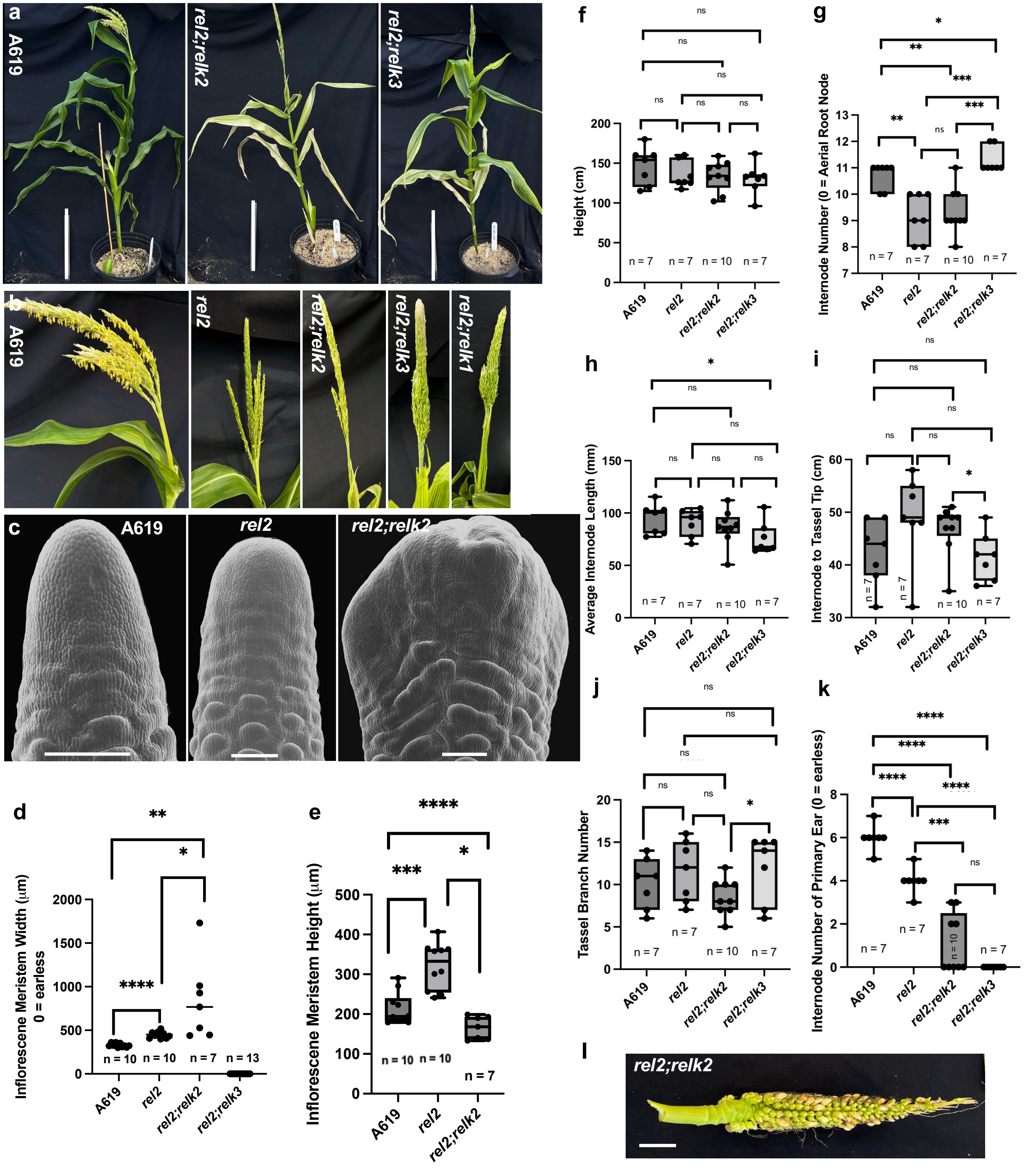

Supplemental Figure S4. Functional redundancy and diversification in *rel2;relk* double mutants. (**a**) Representative whole plant images of A619, *rel2;relk2,* and *rel2;relk3.* Scale bar = 30 cm. Compared to (**b**) A619 tassel and *rel2* tassel, the tassels of *rel2;relk2, rel2;relk3*, and *rel2;relk1* displayed similar genetic enhancement in tassel branch angle. Whereas *rel2;relk2* IM were fasciated (**c-e**), *rel2;relk3* double mutants were predominantly earless. Scale bars = 200µm. In (**c**), SEM images of A619 and *rel2* have been reused from Figure 5a. (**f-k**) Unlike *rel2;relk1,* there were no significant differences in height between A619 and *rel2;relk2* and *rel2;relk3.* Whereas similarly, *rel2;relk2* primary ears were borne on lower internodes relative to *rel2* while *rel2;relk3* was earless. (**l**) Mature ears of *rel2;relk2* are small and produce very few viable seeds. Scale bars = 2.5 cm. Quantification by two-tailed Student's t-test. Scatter plot bold line corresponds to median. Box plot center line corresponds to median; box limits, upper and lower quantiles; whiskers, maximum and minimum values. ns = non-significant ($p > 0.05$), * = $p \leq 0.05$, ** = $p \leq 0.01$, *** $p \leq 0.001$, **** $p \leq 0.0001$.

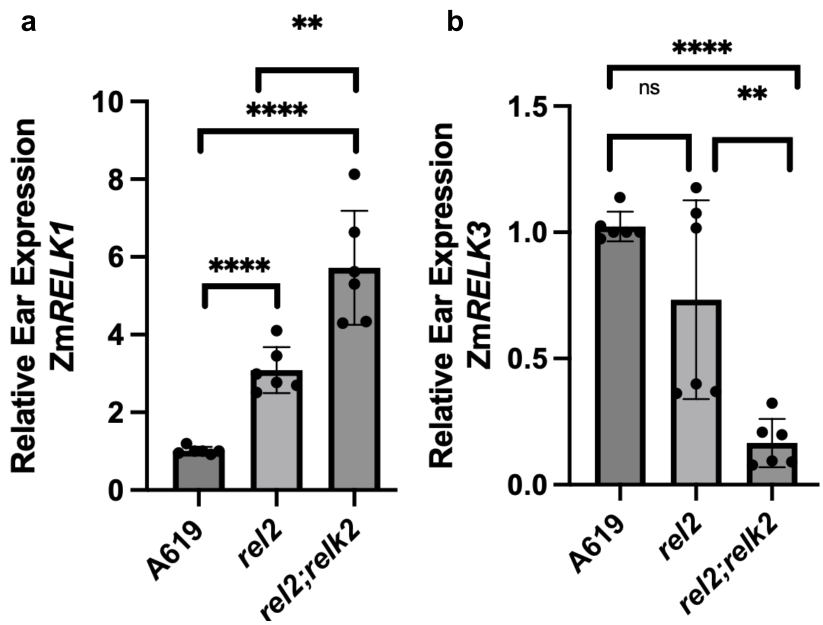

Supplemental Figure S5. *RELK1* but not *RELK3* buffers *rel2;relk2* ear primordia. RT-qPCR of (**a**) *ZmRELK1* and (**b**) *ZmRELK3* expression in 5mm ear primordia of A619, *rel2,* and *rel2;relk2* mutants. two-tailed Student's t-test; n = 3 (pools of three ear samples). Plot displays pooled results from two independent technical replicates. ns = non-significant ($p > 0.05$), * = $p \le 0.05$, ** = $p \le 0.01$, *** $p \le 0.001$, **** $p \le 0.0001$. Error bars, standard deviation.

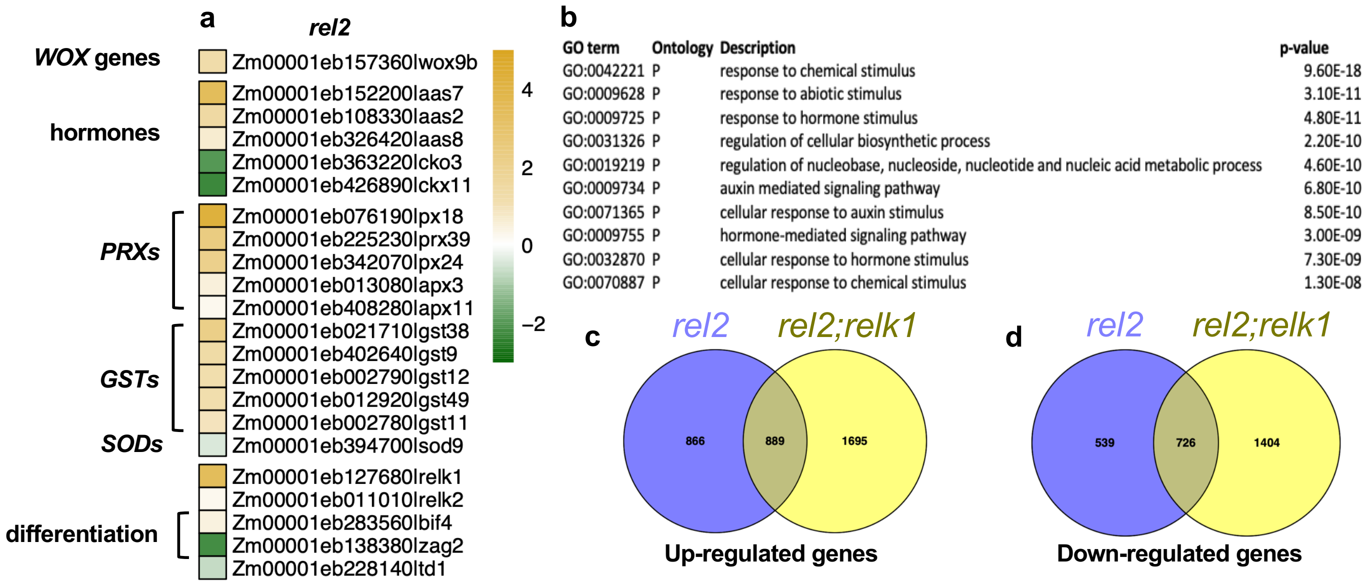

Supplemental Figure S6. Differential gene expression analysis. (**a**) Heat map (scale = logFC) showing mis-regulation of hormone catabolism, ROS scavenging, and differentiations genes in IM tips of *rel2* in line with significantly enriched GO terms (**b**). (**c**) 866 up-regulated and (**d**) 539 down-regulated genes are unique to *rel2,* among these is *RELK1.* ns = non-significant, $p > 0.05$, * = $p \le 0.05$, ** = $p \le 0.01$, *** $p \le 0.001$, **** $p \le 0.0001$.

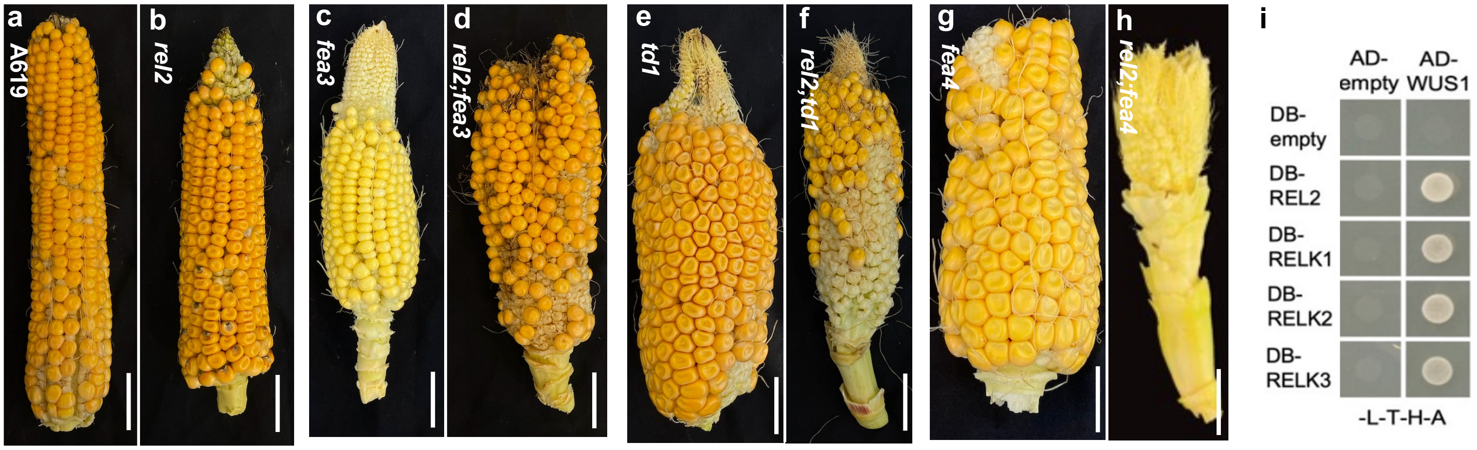

Supplemental Figure S7. Genetic and molecular interactions. Representative images of mature ears of (**a**) A616, (**b**) *rel2*, (**c**) *fea3*, (**d**) *rel2;fea3*, (**e**) *td1,* (**f**) *rel2;td1,* (**g**) *fea4*, (**h**) *rel2;fea4.* Scale bar = 2.5 cm. (**i**) In Y2H assays REL2, RELK1, RELK2, and RELK3 interact with ZmWUS1.

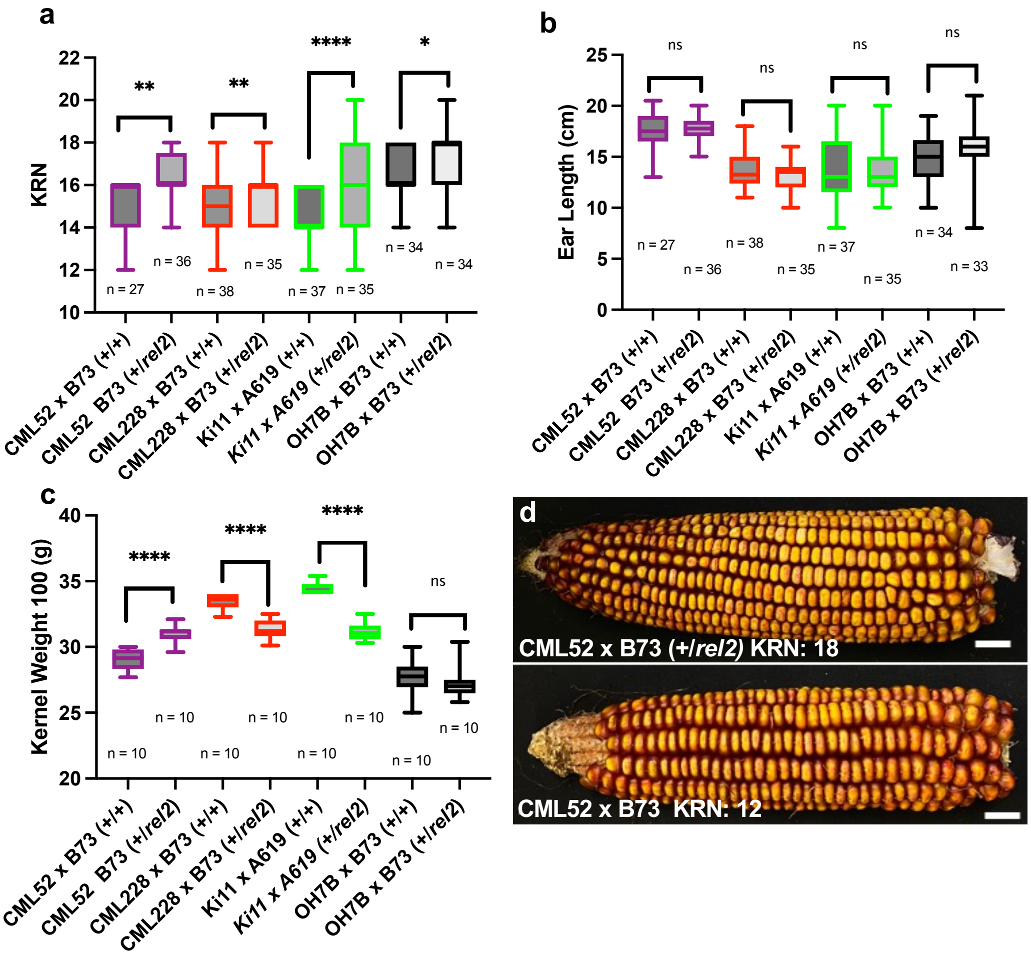

Supplemental Figure S8. Additional NAM F1 Hybrids display Kernel Row Number (KRN) increase. (**a**) 4 additional F1 hybrid combinations displayed a significant increase in KRN; however, only data for one environmental replicate was produced. These combinations displayed non-significant differences in ear length between (+/+) and (+/*rel2*) F1 hybrids (**b**), with pleiotropic effects on kernel weight (**c**). Interestingly, the CML52 x B73 combination produced a significant increase in KRN and kernel weight in the (+/*rel2)* F1 population. (**d**) Representative images of CML52 x B73 (+/+) F1 and (+/*rel2*) F1 ears. Scale bar = 1 cm. Quantification by two-tailed Student's t-test. Box plot center line corresponds to median; box limits, upper and lower quantiles; whiskers, maximum and minimum values. ns = non-significant ($p > 0.05$), * = $p \leq 0.05$, ** = $p \leq 0.01$, *** $p \leq 0.001$, **** $p \leq 0.0001$.
